# A defect in mitochondrial protein translation influences mitonuclear communication in the heart

**DOI:** 10.1038/s41467-023-37291-5

**Published:** 2023-03-22

**Authors:** Feng Gao, Tian Liang, Yao Wei Lu, Xuyang Fu, Xiaoxuan Dong, Linbin Pu, Tingting Hong, Yuxia Zhou, Yu Zhang, Ning Liu, Feng Zhang, Jianming Liu, Andrea P. Malizia, Hong Yu, Wei Zhu, Douglas B. Cowan, Hong Chen, Xinyang Hu, John D. Mably, Jian’an Wang, Da-Zhi Wang, Jinghai Chen

**Affiliations:** 1 Department of Cardiology, Provincial Key Lab of Cardiovascular Research, Second Affiliated Hospital, Zhejiang University School of Medicine, Hangzhou, 310009 China; 2 Institute of Translational Medicine, Zhejiang University School of Medicine, Hangzhou, 310029 China; 3 Department of Cardiology, Boston Children’s Hospital, Harvard Medical School, 300 Longwood Avenue, Boston, MA 02115 USA; 4 Vascular Biology Program, Boston Children’s Hospital, Harvard Medical School, 300 Longwood Avenue, Boston, MA 02115 USA; 5 Department of Clinical Pharmacy, the First Affiliated Hospital, Zhejiang University School of Medicine, Hangzhou, Zhejiang 310003 China; 6 Center for Regenerative Medicine, University of South Florida Health Heart Institute, Morsani School of Medicine, University of South Florida, Tampa, FL 33602 USA; 7 Present Address: Vertex pharmaceuticals, VCGT, 316-318 Northern Ave, Boston, MA 02210 USA

**Keywords:** Gene expression, Organogenesis, Mitochondria, Cardiovascular diseases

## Abstract

The regulation of the informational flow from the mitochondria to the nucleus (mitonuclear communication) is not fully characterized in the heart. We have determined that mitochondrial ribosomal protein S5 (MRPS5/uS5m) can regulate cardiac function and key pathways to coordinate this process during cardiac stress. We demonstrate that loss of *Mrps5* in the developing heart leads to cardiac defects and embryonic lethality while postnatal loss induces cardiac hypertrophy and heart failure. The structure and function of mitochondria is disrupted in *Mrps5* mutant cardiomyocytes, impairing mitochondrial protein translation and OXPHOS. We identify *Klf15* as a *Mrps5* downstream target and demonstrate that exogenous *Klf15* is able to rescue the overt defects and re-balance the cardiac metabolome. We further show that *Mrps5* represses *Klf15* expression through c-myc, together with the metabolite L-phenylalanine. This critical role for *Mrps5* in cardiac metabolism and mitonuclear communication highlights its potential as a target for heart failure therapies.

## Abstract (web summary)

The heart requires high levels of mitochondria to sustain function, and mitochondrial stressors can be transmitted to the nucleus and reprogram metabolism. Here, the authors show that a mitochondrial ribosomal protein is important for heart development in mice by increasing nuclear Klf15 expression.

## Introduction

Mitochondria are known as the powerhouse of the cell; in mature cardiomyocytes, continuous ATP production via oxidative metabolism in the mitochondria is essential for maintaining normal heart function. The mammalian mitochondrion is a semi-autonomous organelle constructed from proteins encoded by both nuclear DNA (nDNA) and mitochondrial DNA (mtDNA); over 1000 nDNA-encoded proteins are localized to mitochondria^1^, while mtDNA encodes 13 proteins that form essential components of the electron transport chain (ETC) involved in the oxidative phosphorylation (OXPHOS) system for ATP generation^2^. Mitochondrial ribosomal proteins (MRPs), required for mammalian mitochondrial translation, are encoded by nDNA^3^, translated by the cytosolic ribosome complex, and then translocated to mitochondria. Deficiency in human MRPs have been linked to forms of cardiovascular disease, cancer, developmental and neurodegenerative disorders, mitochondrial respiratory chain diseases, obesity, and inflammatory disorders^4,5^; specifically, previous reports have established that decreased levels of MRPS6, MRPS10, and MRPS22 are associated with cardiac disorders^6,7^. Mitoribosomal protein MRPS5, also known as uS5m^8^, is a key component of the mitochondrial translational machinery that is highly conserved across species. Mutations in *Mrps5* affect the accuracy of mitochondrial ribosomal translation^9^, establishing a role for MRPS5 in mitochondrial protein translation^10,11^; a decrease in the levels of the mitochondrial protein mt-CO1 were detected in this analysis. In addition, the function of *Mrps5* is linked to cellular stress responses and the reduction of MRPS5 protein levels is also associated with increased longevity in worms and mice^10,11^. Loss of *Mrps5* results in developmental defects that are proposed to result from decreased energy (ATP) production^3^; the decreased ATP pool would be particularly detrimental to the development of organs such as the heart and skeletal muscle, which typically have high energy demands. It is not yet clear whether the translation of mtDNA genes is affected during hypertrophy, but this seems likely given that MRPS5 had been demonstrated to be an important component of the mRNA entry channel in the 28 S subunit of the mammalian mitochondrial ribosome^12,13^; mutations in MRPS5 has been shown to decrease mitochondrial ribosomal translational accuracy in vivo^9^. However, no mechanism has been defined to explain how *Mrps5*-dependent protein translation in the mitochondria is able to transduce changes in cardiac gene expression and metabolism in response to stress.

More than 95% of ATP consumed in the heart is derived from OXPHOS in the mitochondria^14^. Mitochondrial dysfunction triggers a wide variety of pathological processes involved in cardiovascular disease and it is estimated that more than 50% of individuals with mutations in genes encoding mitochondrial proteins progress to some form of cardiomyopathy^15^. Previous studies have demonstrated that cardiac hypertrophy impacts energy generation by mitochondria; it has been found that during cardiac hypertrophy, both nDNA and mtDNA-encoded mitochondrial genes show changes in transcript levels; this results in decreased mitochondrial biogenesis, increased reactive oxygen species (ROS) production, and impaired mitochondrial function^16^.

In this study, we report a critical role for *Mrps5* in heart development, pathological cardiac hypertrophy, cardiac metabolism, and mitonuclear communication. We found that cardiac-specific deletion of *Mprs5* resulted in stalling of mitochondrial ribosomal translation, cristae structure collapse, and subsequent mitochondrial dysfunction. *Mrps5* deficiency links the mitochondrial cristae defect to abnormal cardiac development, pathological cardiac hypertrophy, and heart failure. The use of an AAV9 system to express downstream target genes of MRPS5 in *Mrps5* null hearts resulted in a functional rescue. We further identified the transcription factor Klf15 as a key downstream target in the heart, and that overexpression of *Klf15* was sufficient to rescue the *Mrps5* loss of function phenotypes in the heart. *Klf15* expression was also able to restore the metabolic profile of *Mrps5* null hearts and reverse the observed pathological elevation in glycolysis/gluconeogenesis and decreased expression levels of mitochondrially encoded genes. These observations suggest a paradigm underlying cardiac hypertrophy, one in which pathological remodeling is driven by reprogramming of the cardiac metabolic profile, resulting from defects in mitochondrial translation.

## Results

### Cardiomyocyte-specific *Mrps5* deletion results in cardiac hypertrophy and heart failure

**Fig. 1:**
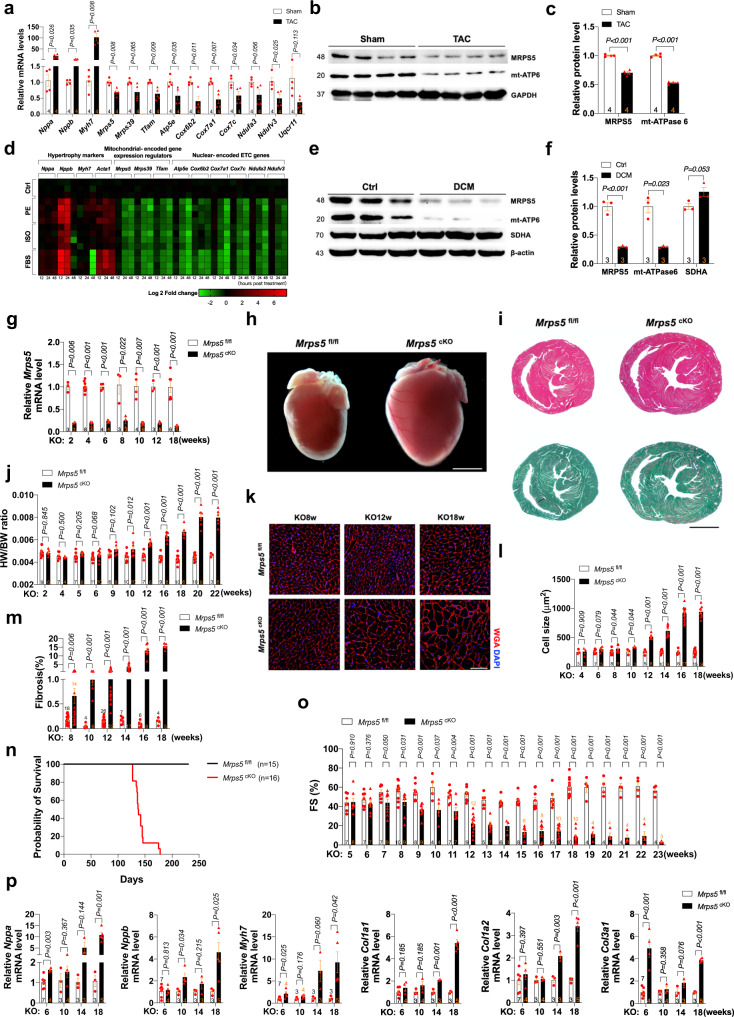
Cardiomyocyte-specific *Mrps5* deletion results in cardiac hypertrophy and heart failure. **a** Gene expression level of *Mrps5* and other regulators of mitochondrial gene expression and ETC genes are downregulated in hypertrophic hearts. **b**, **c** Western blot of protein levels of MRPS5 and mitochondrial encoded protein mt-ATPase 6 in control (Sham) and hypertrophic mouse hearts (TAC). **d** Gene expression level of *Mrps5* and other regulators of mitochondrial gene expression and ETC genes in isolated neonatal mouse cardiomyocytes (NMCMs) in response to hypertrophic agonizts phenylephrine (PE), isoproterenol (ISO) and fetal bovine serum (FBS) in vitro **e**, **f** Analysis of protein levels of MRPS5 and mt-ATPase 6 in human hearts from control or patients diagnosed with dilated cardiomyopathy (DCM). **g** Gene expression level of *Mrps5* is significantly decreased at 2 weeks to 18 weeks after tamoxifen injection. **h** Gross heart morphology from control *Mrps5*^fl/fl^ and *Mrps5*^cKO^ mice at 12 weeks after tamoxifen injection. Scale bar = 500 µm. **i** Heart sections stained with hematoxylin and eosin (top panel) or Sirius Red and Fast Green (bottom panel) from control *Mrps5*^fl/fl^ and *Mrps5*^cKO^ mice at 12 weeks after tamoxifen injection. Scale bar = 500 µm. **j** Heart weight to body weight ratio of *Mrps5*^fl/fl^ and *Mrps5*^cKO^ mice at 2 to 22 weeks after tamoxifen injection. **k** Representative images of heart cross sections from *Mrps5*^fl/fl^ and *Mrps5*^cKO^ mice at 8, 12, and 18 weeks after tamoxifen injection (immunostained with Wheat germ agglutinin (WGA) in red and DAPI in blue). Scale bar = 100 μm. **l** Quantification of the cross-sectional area of cardiomyocytes and **m** Cardiac fibrosis from *Mrps5*^fl/fl^ and *Mrps5*^cKO^ mice at 4 to 18 weeks after tamoxifen injection. **n** Survival curve of *Mrps5*^fl/fl^ and *Mrps5*^cKO^ mice post tamoxifen injection. **o** Echocardiographic measurement of cardiac function in *Mrps5*^fl/fl^ and *Mrps5*^cKO^ mice at 5 to 23 weeks after tamoxifen injection. **p** qRT-PCR analysis of cardiomyopathy marker genes from control *Mrps5*^fl/fl^ and *Mrps5*^cKO^ mouse hearts at 6 to 18 weeks after tamoxifen injection. *N* numbers are indicated in each panel. All data were presented as mean ± SEM. *P* values were determined by a two-tailed unpaired Students’ *t*-test.

To characterize the expression and function of *Mrps5* in cardiomyopathy, we first examined its expression during cardiac remodeling under stress conditions. The expression of *Mrps5*, together with the mitochondrially encoded gene regulators and nuclear-encoded ETC genes (*Atp5e, Cox6b2, Cox7a1, Ndufa3, Ndufv3,* and *Uqcr11*), were all downregulated in a mouse model of pressure overload-induced cardiac hypertrophy and heart failure (transverse aortic constriction, TAC model); the hypertrophic marker genes (*Nppa, Nppb*, and *Myh7*) were upregulated (Fig. 1a–c). Similarly, *Mrps5* expression was significantly downregulated in isolated neonatal mouse cardiomyocytes (NMCMs) in response to the hypertrophic agonizts phenylephrine (PE), isoproterenol (ISO), and fetal bovine serum (FBS), respectively (Fig. 1d). Interestingly, *Mrps5* level was not significantly altered in the heart one month after myocardial infarction (MI); however, its expression was dramatically reduced in the heart six months after MI (Supplementary Fig. 1a, b). Consistent with the above findings in mice, the expression of human MRPS5 was decreased in heart tissue samples from patients with dilated cardiomyopathy (DCM) (Fig. 1e, f, Supplementary Fig. 1c, and Supplementary Table 1).

Next, we sought to determine the functional role of *Mrps5* in the heart in vivo. We used an inducible system to conditionally knockout *Mrps5* (*Mrps5*^cKO^) in adult mouse cardiomyocytes by breeding *Mrps5*^fl/fl^ mice with *αMHC*-MerCreMer transgenic mice (Supplementary Fig. 1d). We confirmed Cre-mediated cardiac-specific deletion of *Mrps5* in the hearts of *αMHC*-MerCreMer; *Mrps5*^fl/fl^ mice (*Mrps5*^cKO^) following tamoxifen administration (Fig. 1g and Supplementary Fig. 1e); no appreciable change in *Mrps5* expression was detected in liver and lung (Supplementary Fig. 1f). Loss of *Mrps5* in the heart results in cardiac hypertrophy, exhibiting as overt enlargement of the heart itself (Fig. 1h, i and Supplementary Fig. g–i) and increased heart weight to body weight ratio (Fig. 1j). Histological examination and quantitative analysis reveal an increase in cardiomyocyte size in *Mrps5*^cKO^ hearts (Fig. 1k, l). Cardiac fibrosis was also substantially increased in the hearts of *Mrps5*^cKO^ mice (Fig. 1m and Supplementary Fig. 1h, i). As a result, *Mrps5*^cKO^ mice began to die after 20 weeks of *Mrps5* ablation, and all mutant mice died within 26 weeks after *Mrps5* ablation (Fig. 1n). Echocardiography was used to carefully monitor the cardiac function of the *Mrps5*^cKO^ and control mice between 5 and 23 weeks after tamoxifen treatment (Supplementary Fig. 1j), and we found that *Mrps5*^cKO^ mice exhibit a decreased fractional shortening (FS%) and ejection fraction (EF%) and increased left ventricular internal diameter (LVID;s) around 8 weeks after *Mrps5* ablation (Fig. 1o and Supplementary Fig. 1j–m). Further evidence of the development of cardiac hypertrophy and cardiomyopathy in *Mrps5*^cKO^ mice includes the upregulation of the hypertrophy and disease marker genes (*Nppa*, *Nppb*, and *Myh7*) and fibrosis marker genes (*Col1a1*, *Col1a2*, and *Col1a3*) in *Mrps5*^cKO^ hearts (Fig. 1p).

### *Mrps5* is required for cardiac development

To gain insight into the function of *Mrps5* during cardiac development, we crossed the *Mrps5*^fl/fl^ mice with cTnT^Cre^ mice, in which the expression of Cre recombinase is under the control of the cardiac promoter of the troponin T2 gene, to generate an early cardiomyocyte specific-targeted *Mrps5* mutant. We analyzed embryos between E10.5 and birth and found that cTnT^Cre^; *Mrps5*^fl/fl^ embryos presented a normal Mendelian ratio at E11.5, but no viable null embryos were found at E12.5 or birth (Supplementary Fig. 2a, b). We examined the embryonic heart at E12.5, prior to the onset of mutant embryo lethality and observed a decrease in myocardial wall thickness (Supplementary Fig. 2c). Transmission electron microscope (TEM) analysis revealed impaired mitochondrial cristae structure in *Mrps5* mutant cardiomyocytes; in addition, the sarcomere structure was affected (Supplementary Fig. 2d). We performed transcriptome sequencing on the *Mrps5* mutant hearts and littermate controls at E12.5 (*n* = 3 biological replicates per group, 5 hearts per replicate) (Supplementary Fig. 2e). Among the dysregulated genes, 1228 genes were upregulated and 930 genes were downregulated (Supplementary Fig. 2f). The enriched pathways based on the KEGG database indicated that cardiac disease associated pathways, including “hypertrophic cardiomyopathy (HCM), dilated cardiomyopathy (DCM) and arrhythmogenic right ventricular cardiomyopathy (ARVC)” were enriched in the *Mrps5* mutant embryonic hearts (Supplementary Fig. 2g). The pathway “regulation of actin cytoskeleton” emerged in the top 20 pathways enriched in the mutant hearts (Supplementary Fig. 2g), consistent with the abnormal sarcomere structure observed via TEM and suggesting that upstream signals contribute to this defect. In addition, enhanced Hippo signaling decreased myogenesis and mitotic spindle genes, which are involved in cardiomyocyte proliferation and myocardial development, likely contributed to the thinner myocardial walls in Mrps5 mutant hearts (Supplementary Fig. 2g, h). Consistent with the observation by TEM that mitochondrial cristae largely collapsed in the Mrps5 mutant hearts, gene set enrichment analysis (GSEA) also showed that oxidative phosphorylation and fatty acid metabolism were significantly reduced. In contrast, glycolysis and reactive oxygen species were enhanced (Supplementary Fig. 2h). These data indicate that *Mrps5* ablation in embryonic hearts results in heart developmental defects that lead to embryonic lethality at E12.5.

### Mitochondrial defects in *Mrps5* mutant hearts

**Fig. 2:**
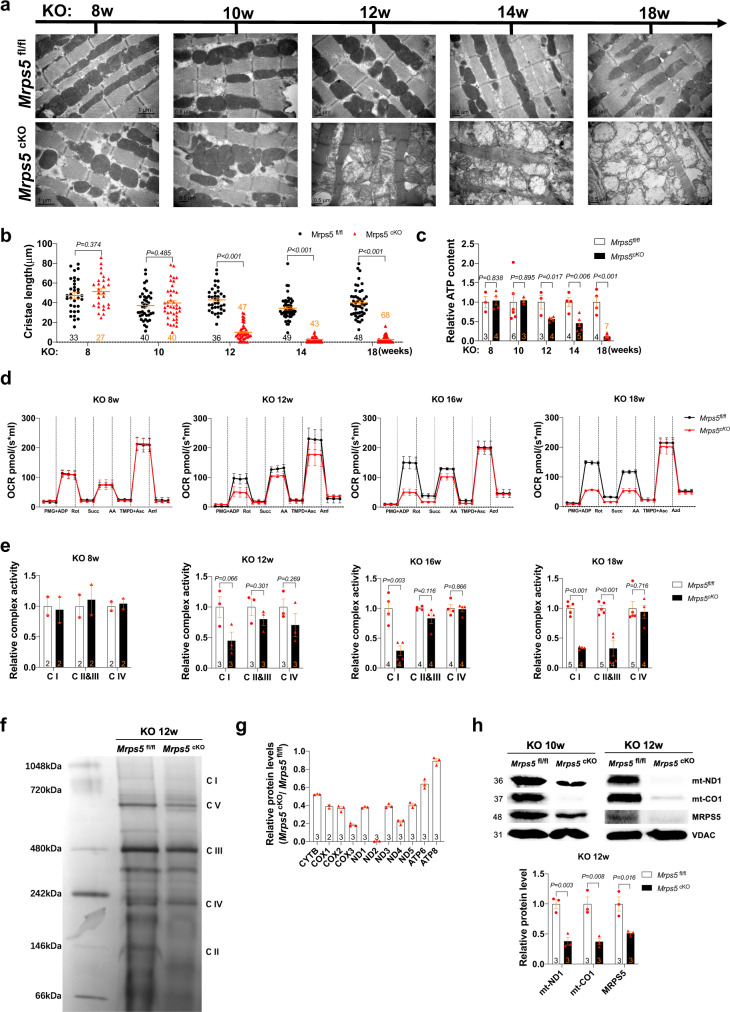
Mitochondrial defects in *Mrps5* mutant hearts. **a** Transmission electronic microscopic (TEM) images of *Mrps5*^fl/fl^ and *Mrps5*^cKO^ hearts at 8 to 18 weeks after tamoxifen injection showing mitochondria (M) and sarcomeres (S). Scale bar as indicated in the bottom of the figure panels. **b** Quantification of mitochondrial cristae length of *Mrps5*^fl/fl^ and *Mrps5*^cKO^ cardiomyocytes from 8 to 18 weeks after tamoxifen injection. **c** Quantification of ATP content in heart tissue samples from *Mrps5*^fl/fl^ and *Mrps5*^cKO^ mice, 8 to 18 weeks after tamoxifen injection. **d** Oxygen consumption rate of *Mrps5*^fl/fl^ and *Mrps5*^cKO^ hearts at 8 to 18 weeks after tamoxifen injection. **e** Quantification of activities of the ETC complexes of *Mrps5*^fl/fl^ and *Mrps5*^cKO^ hearts at 8 to 18 weeks after tamoxifen injection. **f** Immunoblot of mitochondrial electron transport chain protein complexes isolated from *Mrps5*^fl/fl^ and *Mrps5*^cKO^ mouse heart mitochondria at 12 weeks after tamoxifen injection. **g** Quantification of mitochondrial genome encoded protein expression level from *Mrps5*^fl/fl^ and *Mrps5*^cKO^ mouse heart mitochondria via parallel reaction monitoring (PRM) mass spectrometry at 10 weeks after tamoxifen injection. **h** Representative immunoblot and quantification of mitochondrial genome encoded proteins from lysates of *Mrps5*^fl/fl^ and *Mrps5*^cKO^ mouse hearts at 12 weeks after tamoxifen injection. VDAC serves as a loading control. *N* numbers are indicated in each panel. All data were presented as mean ± SEM. *P* values were determined by a two-tailed unpaired Students’ *t*-test.

Organs with a high level of energy consumption, such as the heart, require highly efficient mitochondrial function, which is crucially dependent on mitochondrial OXPHOS. MRPS5 is an important component of the mammalian mitochondrial ribosome^12,13^, and mutant MRPS5 affects mitochondrial ribosomal translational accuracy in vivo^9^; therefore, we sought to define the role of MRPS5 in the mitochondria of cardiomyocytes. We performed a series of the transmission electron microscope (TEM) examinations of heart tissue from *Mrps5*^cKO^ and control *Mrps5*^fl/fl^ mouse cardiomyocytes from 8 weeks to 18 weeks post *Mrps5* ablation (Fig. 2a). No obvious difference in mitochondrial morphology was observed between *Mrps5*^cKO^ and control *Mrps5*^fl/fl^ samples 8–10 weeks after tamoxifen administration (Fig. 2a, b). However, we observed that mitochondrial cristae length drastically decreased after 12 weeks post *Mrps5* ablation; most of the mitochondrial cristae collapsed within 18 weeks of *Mrps5* ablation (Fig. 2a, b).

We performed ATP content assessment in *Mrps5*^cKO^ and *Mrps5*^fl/fl^ hearts. ATP content sharply decreased from 12 weeks post *Mrps5* ablation and was almost undetectable at 18 weeks (Fig. 2c). We measured O_2_ consumption of myofibers isolated from both *Mrps5*^cKO^ and *Mrps5*^fl/fl^ hearts and found a dramatic decrease in O_2_ consumption in *Mrps5*^cKO^ hearts (Fig. 2d). We monitored the activities of mitochondrial ETC complexes and found that they started to decrease 12 weeks post *Mrps5* ablation, decreasing even further by 18 weeks. This is especially the case for complexes I, II, and III (Fig. 2e). Blue native polyacrylamide gel electrophoresis (BNPAGE) analysis confirmed this observation (Fig. 2f). Given the ETC complexes are made up of nDNA- and mtDNA-encoded proteins, we performed parallel reaction monitoring (PRM) to assess the levels of mtDNA-encoded proteins; this analysis revealed a marked reduction in the levels of mtDNA-encoded proteins in the hearts of *Mrps5*^cKO^ mice compared to the controls (Fig. 2g). Western blotting demonstrated that mt-ND1 and mt-CO1 protein levels were substantially decreased in *Mrps5*^cKO^ hearts, confirming that *Mrps5* ablation resulted in impaired mitochondrial translation (Fig. 2h). Together, these studies suggest that loss of *Mrps5* in the heart leads to structural and functional defects in mitochondria.

### Loss of *Mrps5* alters translational and metabolic programs in the heart

**Fig. 3:**
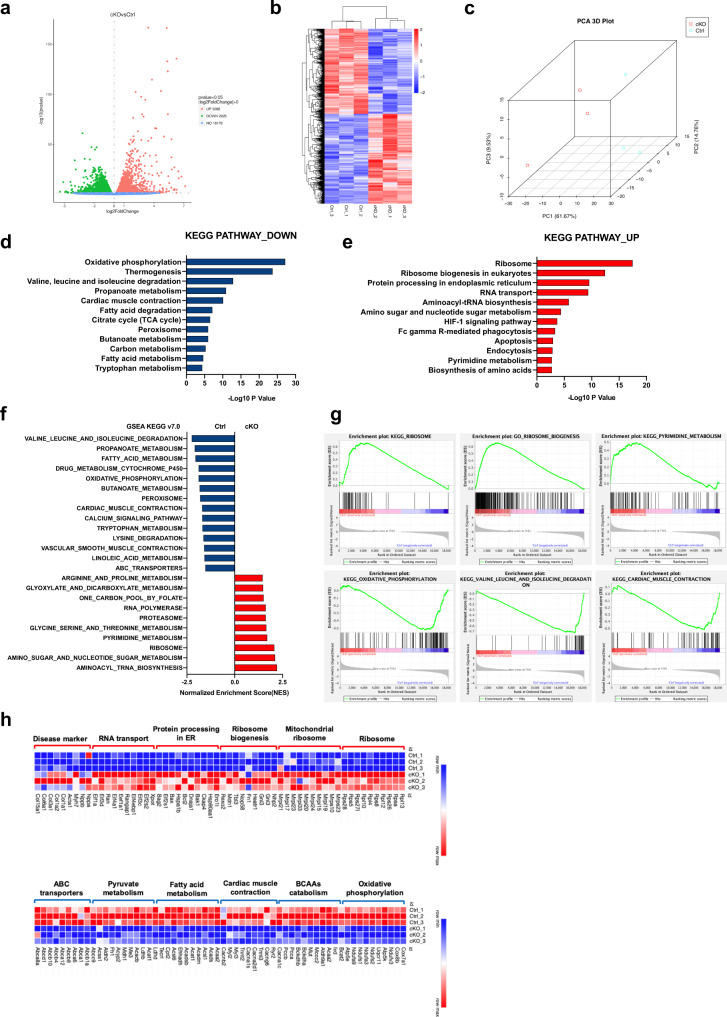
Loss of *Mrps5* alters translational and metabolic programs in the heart. **a** Volcano plot of dysregulated transcripts in *Mrps5*^cKO^ hearts by comparison with *Mrps5*^fl/fl^ hearts at 12 weeks following tamoxifen injection. **b** Hierarchical clustering heatmap of dysregulated transcripts in *Mrps5*^cKO^ hearts. *n* = 3. **c** Principal component analysis (PCA) of gene expression in *Mrps5*^fl/fl^ and *Mrps5*^cKO^ hearts. *n* = 3. **d** Kyoto Encyclopedia of Genes and Genomes (KEGG) functional enrichment analysis of downregulated genes in *Mrps5*^cKO^ hearts. **e** KEGG functional enrichment analysis of the upregulated genes in *Mrps5*^cKO^ hearts. **f** Gene set enrichment analysis (GSEA) showing dysregulated signaling pathways in *Mrps5*^cKO^ hearts. **g** Enrichment plots of key pathways in *Mrps5*^cKO^ hearts. **h** Heatmaps of the relative expression of the differentially expressed genes in dysregulated pathways identified in *Mrps5*^cKO^ hearts. *P* values were determined by two-tailed unpaired Students’ *t*-test in (**a**, **d**, **e**).

To understand the molecular mechanisms underlying *Mrps5* function in the heart, we performed RNA sequencing of *Mrps5*^cKO^ and *Mrps5*^fl/fl^ hearts 12 weeks after tamoxifen induction. We chose this stage because the mutant hearts have just started to exhibit detectable defects, and the changes in the transcriptome state would more accurately correlate with the onset of the phenotype. Volcano plot, heatmap, and principal component analysis (PCA) of the RNA sequencing confirmed the consistency between biological repeats for each condition (Fig. 3a–c). We identified a set of 6014 genes that were differentially expressed in *Mrps5*^cKO^ hearts; 3088 genes are upregulated, and 2926 genes are downregulated (*Mrps5* itself is among the most noticeably downregulated genes) (Fig. 3a). As expected, DAVID tools (Database for Annotation, Visualization, and Integrated Discovery) and KEGG database analyses revealed that oxidative phosphorylation, thermogenesis, branched-chain amino acids (BCAAs) degradation, cardiac muscle contraction, citrate cycle (TCA cycle), and fatty acid metabolism were among the most affected pathways associated with downregulated genes in *Mrps5*^cKO^ hearts (Fig. 3d), consistent with the findings of our phenotypic analyses showing that *Mrps5*^cKO^ hearts display defects in mitochondria and metabolism. Conversely, genes associated with pathways related to the ribosome, ribosome biogenesis, and protein processing in the endoplasmic reticulum are among the most enriched in the hearts *of Mrps5*^cKO^ mice (Fig. 3e). Given the known essential function of MRPS5 in mitochondrial protein translation, these data suggest a compensatory mechanism in *Mrps5*^cKO^ cardiomyocytes to activate the translational program. These findings are further supported by the results of our analysis of differentially expressed genes via the reactome and the gene ontology (GO) biological processes databases (Supplementary Fig. 3a–d). Additional analysis using GSEA confirmed that “ribosome” and “ribosome biogenesis” are among the top pathways associated with the upregulated genes, while “oxidative phosphorylation” and “cardiac muscle contraction” are associated with downregulated genes in *Mrps5*^cKO^ hearts (Fig. 3f, g and supplementary Fig. 3e–g). A heatmap to visualize the expression of representative genes for the significantly altered signaling pathways further confirmed these observations (Fig. 3h). These results support an important role for MRPS5 as a regulator of translation and metabolism in the heart.

### Functional screening identifies Klf15 as an *Mrps5* downstream target in the heart

**Fig. 4:**
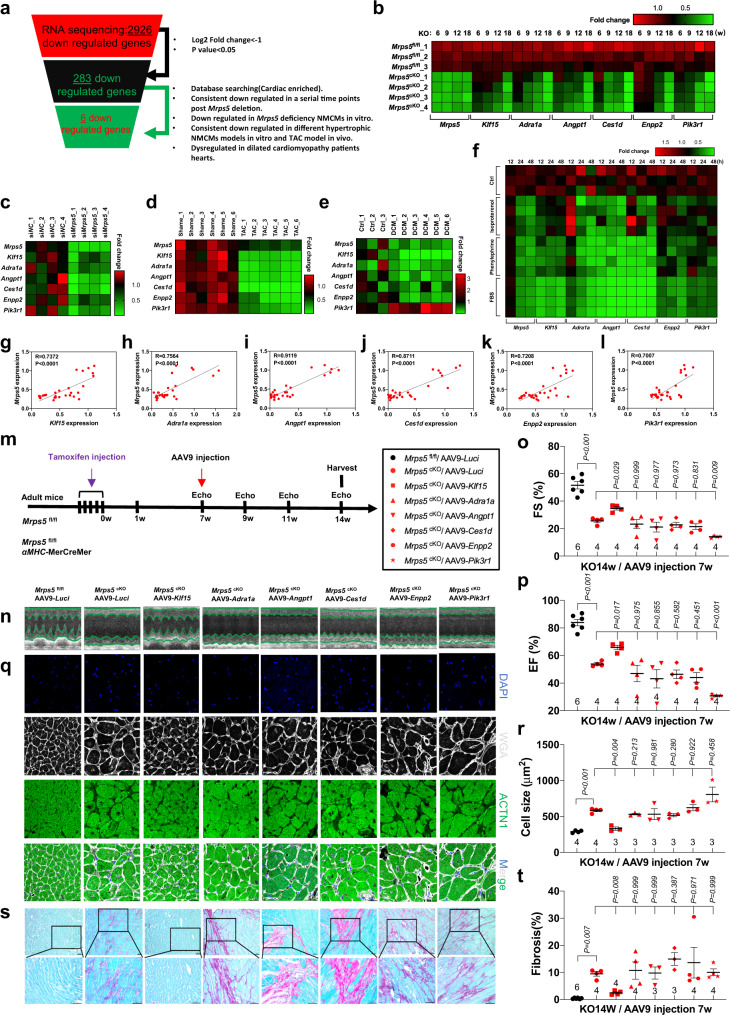
Functional screening identifies KLF15 as an *Mrps5* downstream target in the heart. **a** Diagram of workflow for the selection of six *Mrps5* downstream target genes from the 2926 downregulated genes identified in *Mrps5*^cKO^ hearts. **b** Expression of *Klf15* and other top candidate genes in the hearts of *Mrps5*^fl/fl^ control and *Mrps5*^cKO^ hearts. **c** Decreased expression of *Klf15* and other top candidate genes in *Mrps5* knockdown cardiomyocytes. **d** Heatmap showing expression of *Mrps5*, *Klf15*, and other top candidate genes in TAC-induced hypertrophic mouse hearts. **e** Dysregulated expression of *Mrps5*, *Klf15,* and other top candidate genes in hearts of human dilated cardiomyopathy patients. **f** Heatmap of the relative gene expression for *Mrps5*, *Klf15*, and other top candidate genes in NMCMs after 12, 24, 48 h stimulation with ISO, PE, and FBS, respectively. **g**–**l** Pearson’ *r* correlation coefficient with corresponding *P* values for the covariation between *Mrps5*, *Klf15*, and other top candidate genes. **m** Experimental procedure for the use of AAV9-mediated expression of potential targets for functional screening in *Mrps5*^cKO^ mice. **n** M-mode echocardiography of *Mrps5*^cKO^ mice, 7 weeks after injection of indicated AAV9-construct. **o** Fractional shortening (FS%) of *Mrps5*^cKO^ mice, 7 weeks after injection with indicated AAV9-construct. **p** Ejection fraction (EF) of *Mrps5*^cKO^ mice, 7 weeks after injection with indicated AAV9-construct. **q** Immunohistology of heart sections of *Mrps5*^cKO^ mice, 7 weeks after injection with indicated AAV9-construct. DAPI labels the nucleus, WGA marks the cell membrane, and ACTN1 marks cardiomyocytes. Scale bars = 20 µm. **r** Quantification of sizes of cardiomyocytes from the previous experiment (Fig. 4q). **s** Sirius Red and Fast Green staining of heart sections of *Mrps5*^cKO^ mice, 7 weeks after injection of indicated AAV9-construct. Scale bars = 50 µm. The boxed area in the upper panel is shown magnified in the lower panel. **t** Quantification of fibrosis from the previous experiment (Fig. 4s). *N* numbers are indicated in each panel. All data were presented as mean ± SEM. *P* values were determined by one-way ANOVA with the Brown–Forsythe and Welch multiple comparisons test.

We reasoned that genes downregulated in *Mrps5*^cKO^ hearts would be good candidates for mediators of the translational machinery collapse observed upon *Mrps5* ablation. We used multiple criteria to prioritize genes for investigation from 2926 that were downregulated in *Mrps5*^cKO^ hearts (Fig. 4a). The gene expression data derived from the RNA-sequencing dataset was subjected to further analysis and experimentation, then sorted as follows; (1) expression decrease greater than 50% (283 genes), (2) genes exhibiting cardiac enriched expression patterns (60/283), (3) genes downregulated in a continuous pattern following *Mrps5* ablation (18/60), (4) genes consistently downregulated upon hypertrophic stimuli in vitro and in vivo, established through experimentation (12/18), (5a) downregulation upon knockdown of *Mrps5* in neonatal mouse cardiomyocytes (NMCMs), and (5b) genes consistently downregulated in the hearts of patients with dilated cardiomyopathy (DCM) (6/12). From this stepwise screening, we prioritized six genes for analysis: *Klf15 (*Kruppel-like factor 15, *Klf15*), *Adra1a (*Adrenergic receptor, alpha 1α, and *Adra1a*), *Angpt1 (*Angiopoietin 1 and *Angpt1*), *Ces1d* (Carboxylesterase 1D and *Ces1d*), *Enpp2* (ectonucleotide pyrophosphatase/phosphodiesterase 2, *Enpp2*), and *Pik3r1* (phosphoinositide-3-kinase regulatory subunit 1, *Pik3r1*).

The expression of these six genes is significantly reduced in heart tissue at 6, 9, 12, and 18 weeks post *Mrps5* ablation (Fig. 4b); these observations were confirmed in NMCMs using siRNA to knockdown *Mrps5* (Fig. 4c). The expression of *Mrps5* and these six genes is similarly reduced in TAC-induced hypertrophic mouse hearts (Fig. 4d). Interestingly, all but one of these genes are also decreased in the hearts of human patients with DCM; the exception is *Pik3r1*, which displayed an increased expression pattern when compared to control patient samples (Fig. 4e). We further examined the expression pattern of these six candidate genes in models of cardiomyocyte stress in vitro; NMCMs were treated with isoproterenol (ISO), phenylephrine (PE) and fetal bovine serum (FBS) stimulation for 12, 24, and 48 h (Fig. 4f). Expression of all six genes was reduced and correlated with decreased expression of *Mrps5* (Fig. 4g–l). The shared expression responses of these six candidate genes upon decreased levels of *Mrps5* suggest that they may mediate the function of *Mrps5* in the heart.

Next, we asked whether the introduction of each of these candidate genes into *Mrps5*^cKO^ mice could rescue the associated cardiac defects. We also included *Pik3r1* in these experiments to determine if it had any influence, since the expression of this gene was increased in DCM patients (instead of decreased, like the others). We employed the well-established AAV9 gene delivery system for neonatal and adult mice with the noted modifications and found that GFP was easily detected in mouse hearts after AAV9-*Gfp* injection, as we have previously reported (Supplementary Fig. 4a–f).

We constructed cardiac-specific AAV9-*Klf15*, AAV9-*Adra1a*, AAV9-*Angpt1*, AAV9-*Ces1d*, AAV9-*Enpp2*, AAV9-*Pik3r1*, and control AAV9-*Luci* viruses, and injected them into *Mrps5*^cKO^ or *Mrps5*^fl/fl^ mice 7 weeks after the final tamoxifen injection. Expression of these target genes in the heart was confirmed (Supplementary Fig. 4g–l). We evaluated cardiac function in these mice using echocardiography 2, 4, and 7 weeks after the AAV injection (Fig. 4m and Supplementary Fig. 5a). Seven weeks after *Mrps5* ablation and before the AAV9 treatment, there were no differences in heart function between the groups of *Mrps*5^cKO^ mice, though they showed a slight reduction in cardiac function when compared with control *Mrps5*^fl/fl^ mice (Supplementary Fig. 5b–e). Nine weeks post *Mrps5* ablation (2 weeks post AAV9 infection), the cardiac function of *Mrps5*^cKO^ mice treated with the control AAV9-*Luci* virus significantly decreased compared to that of the *Mrps5*^fl/fl^ mice. However, AAV9-*Klf15* and AAV9-*Adra1a* treatment preserved cardiac function in *Mrps5*^cKO^ mice compared to control AAV9-*Luci*-treated animals. The introduction of *Angpt1*, *Ces1d*, and *Enpp2* expression displayed modest cardiac protection in *Mrps5*^cKO^ mice. In contrast, AAV-*Pik3r1* failed to rescue cardiac function in *Mrps5*^cKO^ mice (Supplementary Fig. 5a, f–i). Eleven weeks post *Mrps5* ablation (4 weeks post AAV9 infection), AAV9-delivered *Klf15* and *Adra1a* continued to rescue heart function in *Mrps5*^cKO^ mice while *Angpt1*, *Ces1d*, and *Enpp2* showed diminished protection in *Mrps5*^cKO^ mice; AAV9-*Pik3rl* treatment worsened the cardiac function of *Mrps5*^cKO^ mice compared to AAV9-*Luci* control (Supplementary Fig. 5a, j–m). Finally, 14 weeks post *Mrps5* ablation (7 weeks post AAV9 infection), only AAV9-*Klf15* treatment continued to ameliorate the cardiac phenotype of *Mrps5*^cKO^ mice (Fig. 4n–p and Supplementary Fig. 5n, o). The other candidate genes (*Adra1a*, *Angpt1*, *Ces1d*, and *Enpp2)* did not maintain their beneficial effects in the heart of *Mrps5*^cKO^ mice and, as predicted, AAV9-*Pik3r1* furtherly impaired heart function in *Mrps5*^cKO^ mice (Fig. 4o, p and Supplementary Fig. 5n).

We further investigated whether the reintroduction of the identified candidate genes could attenuate the massive cardiac hypertrophy that develops in *Mrps5*^cKO^ mice. While AAV9-*Klf15* treatment significantly reduced hypertrophy in *Mrps5*^cKO^ mouse hearts, overexpression of *Adra1a*, *Angpt1*, *Ces1d*, and *Enpp2* failed to do so; consistent with the previous observations, AAV9-*Pik3r1* treatment resulted in a further increase in hypertrophy in *Mrps5*^cKO^ mice (Fig. 4q, r). Similarly, only overexpression of AAV9-*Klf15* was able to prevent cardiac fibrosis in the hearts of *Mrps5*^cKO^ mice (Fig. 4s, t). Finally, we examined the molecular signatures for cardiac hypertrophy and fibrosis in *Mrps5*^cKO^ hearts after overexpression of these 6 candidate genes. Our results revealed that only AAV9-*Klf15* treatment reduced the expression of the hypertrophic marker gene *Nppa* and fibrotic marker gene *Col1a1* in *Mrps5*^cKO^ hearts (Supplementary Fig. 5p–r). These data strongly support *Klf15* as a gene that can mediate the rescue of cardiac defects in *Mrps5*^cKO^ mice.

### Overexpression of *Klf15* prevents and rescues cardiac defects and the progression of cardiac hypertrophy in *Mrps5* mutant mice

**Fig. 5:**
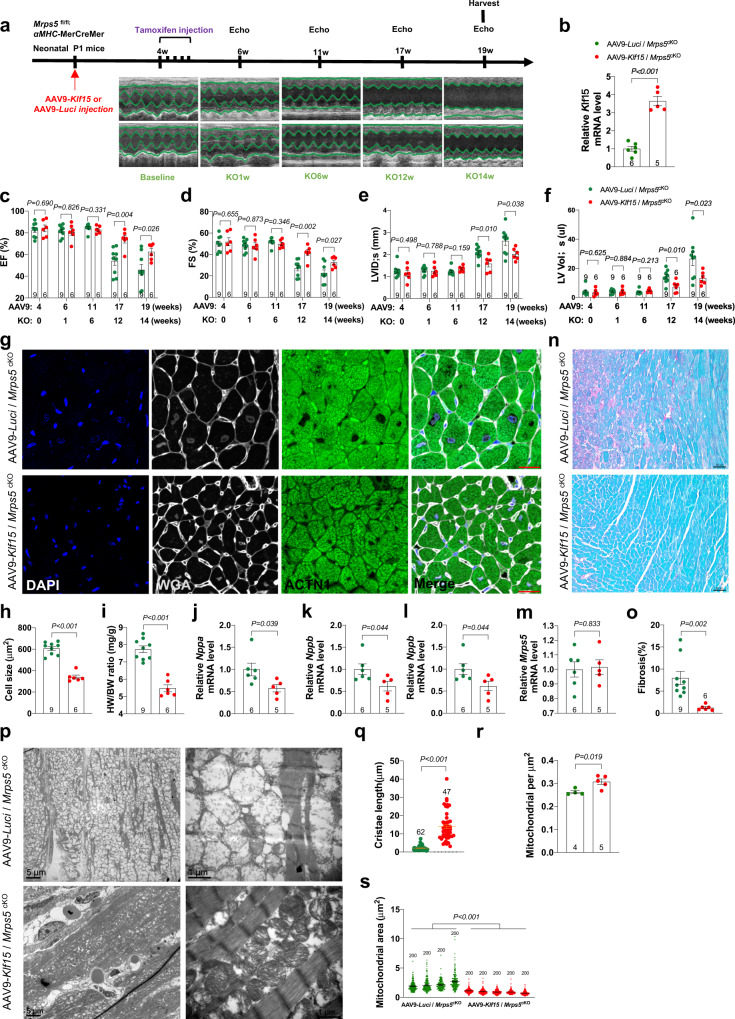
Overexpression of KLF15 prevents cardiac defects in *Mrps5* mutant mice. **a** Experimental procedure to test the function of *Klf15* in preventing the development of cardiomyopathy in *Mrps5*^cKO^ mice. Neonatal *Mrps5*^cKO^ mice were injected with control AAV9-*Luci* or AAV9-*Klf15*. Tamoxifen was injected at 4 weeks to induce the deletion of *Mrps5* in the heart. Echocardiography was performed at indicated time points (M-mode data shown). **b** Gene expression of *Klf15* in *Mrps5*^cKO^ mice injected with AAV-*Luci/Klf15*, respectively. **c**–**f** Ejection fraction (EF), fractional shortening (FS), left ventricular systolic diameter (LVID;s), and left ventricular volume (LV Vol;s) were recorded in AAV9-*Luci* or AAV9-*Klf15* injected *Mrps5*^cKO^ mice at indicated time points. **g** Immunohistology of heart sections of *Mrps5*^cKO^ mice after neonatal injection of AAV9-*Luci* or AAV9-*Klf15*. DAPI (nucleus), WGA (cell membrane), and ACTN1 (cardiomyocytes). Scale bars = 20 µm. **h** Quantification of cardiomyocyte size from Fig. 5g. **i** Heart weight to body weight ratio of AAV9-*Luci* or AAV9-*Klf15* injected *Mrps5*^cKO^ mice. **j**–**m** qRT-PCR analysis of *Nppa*, *Nppb*, *Acta1*, and *Mrps5* genes in 19-week-old *Mrps5*^cKO^ hearts after neonatal injection of AAV9-*Luci* or AAV9-*Klf15*. **n** Sirius Red and Fast Green staining of heart sections from *Mrps5*^cKO^ mice at 19 weeks of age following neonatal injection of AAV9-*Luci* or AAV9-*Klf15*. Scale bars = 50 µm. **o** Quantification of fibrosis from hearts described in Fig. 5n. **p** TEM images of heart tissue from 19-week-old *Mrps5*^cKO^ mice injected at the neonatal stage with AAV9-*Luci* or AAV9-*Klf15*. Scale bars as indicated. **q** Quantification of mitochondrial cristae length from hearts of mice described in Fig. 5p. **r** Quantification of mitochondrial numbers per µm^2^ in heart tissue from mice described in Fig. 5p. **s** Quantification of the mitochondrial area in heart tissue from mice described in Fig. 5p. **h**–**o**, **q**–**s** Comparisons between *Mrps5*^cKO^ mice injected with AAV9-*Luci* (green) or AAV9-*Klf15* (red), as indicated in the legend for (Fig. 5r). *N* numbers are indicated in each panel. All data were presented as mean ± SEM. *P* values were determined by a two-tailed unpaired Students’ *t*-test.

We asked whether neonatal overexpression of *Klf15 in Mrps5*^cKO^ mice, before any phenotypic observations could prevent the onset of cardiac defects in adulthood. AAV9-*Klf15* or control AAV9-Luci were injected into *Mrps5*^fl/fl^;*α*MHC-MerCreMer mice at postnatal day 1 (P1), tamoxifen was administrated 4 weeks later to induce the deletion of the *Mrps5 gene*, and cardiac function was monitored at set time points after *Mrps5* ablation (Fig. 5a). We confirmed the efficacy of AAV9-mediated overexpression of *Klf15* in the hearts of *Mrps5*^cKO^ mice (Fig. 5b). No obvious differences in cardiac function between AAV9-*Klf15* and AAV9-*Luci* treated *Mrps5*^cKO^ groups were observed at 6 weeks (Fig. 5c–f). By 12 weeks post *Mrps5* ablation, AAV9-*Luci* injected control *Mrps5*^cKO^ mice exhibited a dramatic decrease in cardiac function, similar to what was observed in the untreated mice with deletion of the *Mrps5* gene in the heart; however, AAV9-*Klf15* treatment substantially improved cardiac function as indicated by the improved ejection fraction (EF%), fractional shortening (FS%), decreased left ventricular internal diameter at the end of systole (LVID;s), and left ventricular volume at the end of systole (LV Vol;s) (Fig. 5c–f). AAV9-*Klf15* treatment also repressed cardiac hypertrophy, as demonstrated by the reduction in heart weight/body weight ratio and cardiomyocyte size (Fig. 5f–i). Consistently, the expression of the hypertrophic marker genes, *Nppa*, *Nppb*, and *Acta1* was suppressed in AAV9-*Klf15* treated *Mrps5*^cKO^ mice hearts compared to the control group (Fig. 5j–m). Furthermore, *Klf15* overexpression in *Mrps5*^cKO^ mice hearts significantly suppressed cardiac fibrosis (Fig. 5n, o). One of the most dramatic defects in the *Mrps5*^cKO^ hearts is the disruption of the mitochondria (Fig. 2a). Therefore, we investigated whether *Klf15* overexpression was sufficient to preserve the structure of mitochondria in *Mrps5*^cKO^ hearts. Indeed, mitochondrial cristae structure was improved, with most remaining intact, in AAV9-*Klf15* treated hearts while control AAV-Luci treated hearts displayed mitochondrial cristae disruption (Fig. 5p). This observation is further supported by quantification of the length of cristae, the mitochondria number, and area. We found that re-expression of Klf15 results in an increase in mitochondrial cristae and mitochondrial numbers, whereas mitochondrial area decreased (Fig. 5q–s). In addition to these improvements in mitochondrial morphology and number, we observed a restoration in the levels of mitochondrial ETC proteins in the *Mrps5*^cKO^ hearts after the re-expression of Klf15 (Supplementary Fig. 6).

**Fig. 6:**
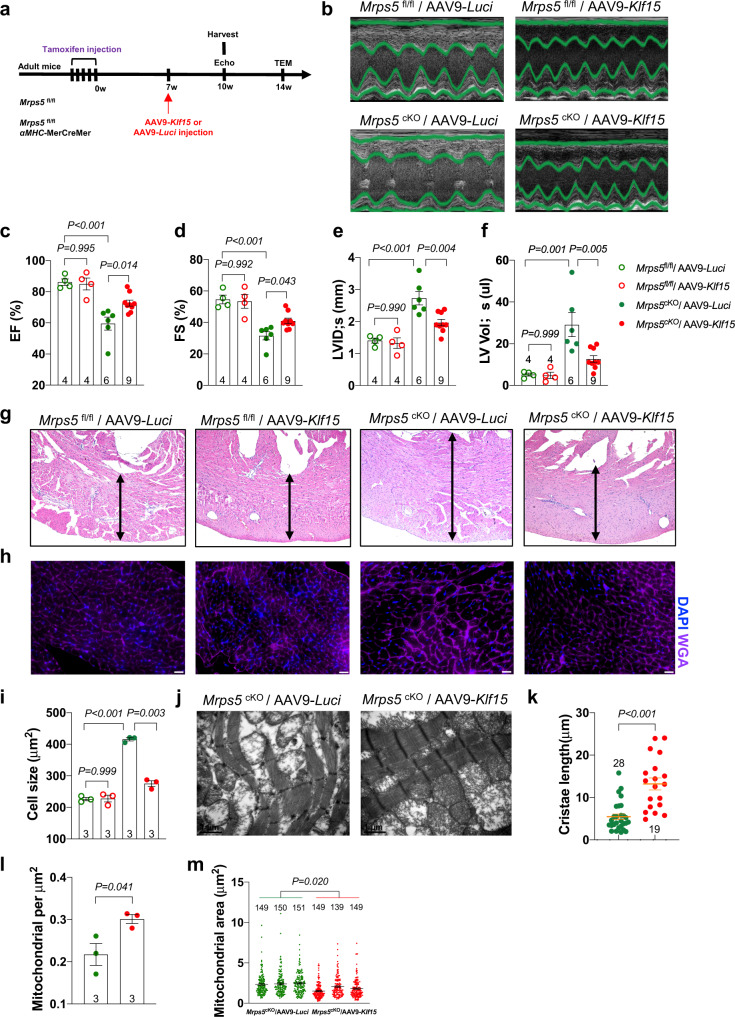
Restoration of cardiac *Klf15* expression rescues heart defects in *Mrps5*^cKO^ mutant mice. **a** Schematic depiction of approach used for delivery of AAV9- *Luci*/*Klf15* to the adult *Mrps5*^fl/fl^/*Mrps5*^cKO^ mouse hearts. **b** Representative examples of M-mode echocardiography recorded at 10 weeks after tamoxifen injection of *Mrps5*^fl/fl^ and *Mrps5*^cKO^ mice injected with either AAV9-*Luci* or AAV9-*Klf15* (as indicated). **c**–**f** Quantification of EF, FS, LVID;s, and LV Vol;s from each group as indicated in legend for **f**, at 10 weeks after tamoxifen injection. **g** Representative images of H&E stained cross sections of heart tissue from each group as indicated. **h** Representative images of cross sections of heart tissue from each group as indicated. The heart sections were immunostained with WGA in purple and DAPI in blue. Scale bar = 25 μm. **i** Quantification of the cross-sectional area of cardiomyocytes from hearts of mice at 10 weeks after tamoxifen injection for each group as indicated in legend for (Fig. 6f). **j** TEM images of AAV9-*Luci* or AAV9-*Klf15* injected *Mrps5*^cKO^ mice at 14 weeks post last tamoxifen injection. Scale bar as indicated in panels. **k** Quantification of mitochondrial cristae length from AAV9-*Luci* or AAV9-*Klf15* injected *Mrps5*^cKO^ mice described in Fig. 6j. **l** Quantification of mitochondrial numbers per μm^2^ from AAV9-*Luci* or AAV9-*Klf15* injected *Mrps5*^cKO^ mice described in Fig. 6j. **m** Quantification of the mitochondrial area from AAV9-*Luci* or AAV9-*Klf15* injected *Mrps5*^cKO^ mice described in Fig. 6j. *N* numbers are indicated in each panel. All data are presented as mean ± SEM. *P* values were determined by one-way ANOVA with the Brown–Forsythe and Welch multiple comparisons test.

Encouraged by the findings that overexpression of *Klf15* in neonatal *Mrps5*^cKO^ mice was sufficient to prevent the development of cardiac hypertrophy, we explored the potential for *Klf15* in the treatment of cardiac defects in adult Mrps5 mutant mice. *Mrps5*^fl/fl^ mice were included as a control to ensure that overexpression of *Klf15* alone did not result in an irregular phenotype in normal mice (Fig. 6a). Similar to what we reported previously, AAV9-mediated overexpression of Klf15 preserved cardiac function (Fig. 6b–f) and repressed cardiac hypertrophy (Fig. 6g–i). In AAV9-*Klf15* treated *Mrps5*^cKO^ hearts, mitochondrial cristae structure was well preserved, and we also observed increased mitochondrial numbers and decreased mitochondrial area (Fig. 6j–m). In contrast, control AAV9-Luci failed to rescue the cardiac defects in Mrps5 mutant mice (Fig. 6b–k). Significantly, overexpression of *Klf15* did not seem to cause any defect in control *Mrps5*^fl/fl^ mice (Fig. 6b–i).

### *Klf15* regulates cardiac metabolism and rescues the imbalanced metabolome in *Mrps5* mutant hearts

**Fig. 7:**
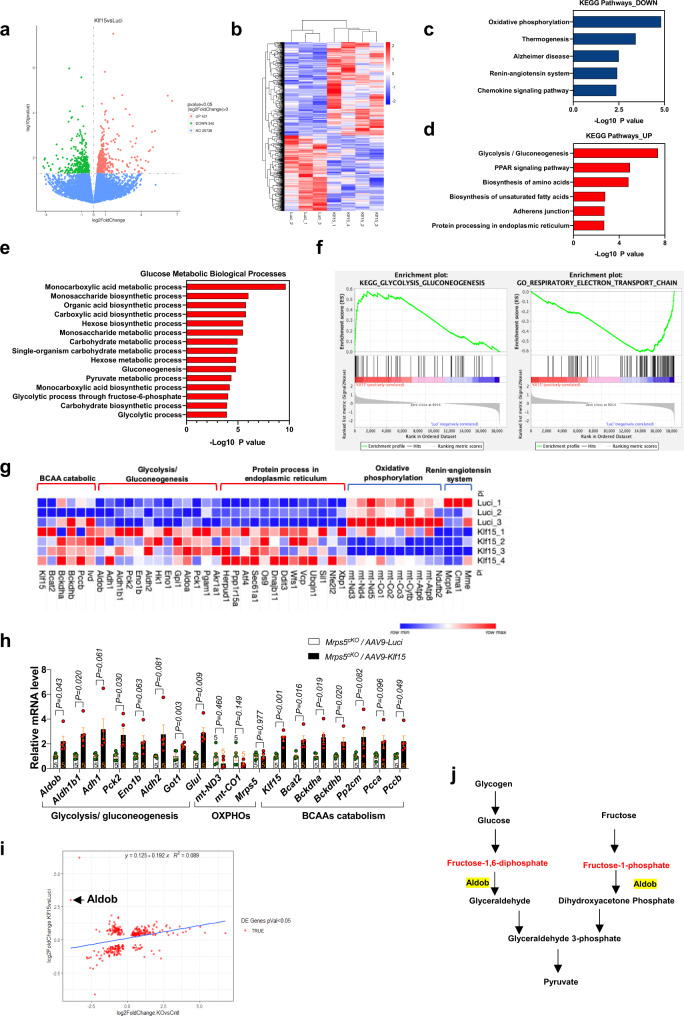
Klf15 regulates cardiac metabolism and corrects imbalanced metabolome in *Mrps5* mutant hearts. **a** Volcano plot of the dysregulated genes in *Mrps5*^cKO^/AAV-*Klf15* by comparison with *Mrps5*^cKO^/AAV-*Luci* hearts. **b** Hierarchical clustering heatmap of dysregulated genes in *Mrps5*^cKO^/AAV-*Luci* and *Mrps5*^cKO^/AAV-*Klf15* hearts. **c** Kyoto Encyclopedia of Genes and Genomes (KEGG) functional enrichment analysis of the downregulated genes in *Mrps5*^cKO^/AAV-*Klf15* hearts. **d** KEGG functional enrichment analysis of the upregulated genes in *Mrps5*^cKO^/AAV-*Klf15* hearts. **e** Gene Ontology (GO) analysis of the glucose metabolic biological processes affected in *Mrps5*^cKO^/AAV-*Klf15* hearts. **f** Heatmaps of the relative expression of the differentially expressed genes in dysregulated pathways identified in *Mrps5*^cKO^/AAV-*Luci* and *Mrps5*^cKO^/AAV-*Klf15* hearts. **g** Gene set enrichment analysis (GSEA) showing dysregulated signaling pathways in *Mrps5*^cKO^/AAV-*Klf15* hearts. **h** qRT-PCR analysis of the mRNA expression level of glycolysis/gluconeogenesis, OXPHOS, and BCAAs catabolic associated genes in *Mrps5*^cKO^/AAV-*Luci* (*n* = 5) and *Mrps5*^cKO^/AAV-*Klf15* hearts (*n* = 5). **i** Scatter diagram of dysregulated genes in *Mrps5*^cKO^ versus *Mrps5*^fl/fl^ hearts and *Mrps5*^cKO^/AAV-*Klf15* versus *Mrps5*^cKO^/AAV-*Luci* hearts. The gene expression level of *Aldob* is reduced in *Mrps5*^cKO^ but enhanced in *Mrps5*^cKO^/AAV-*Klf15* hearts. **j** Diagram illustrating the position and role of *Aldob* in the glycolysis/gluconeogenesis pathway. All data were presented as mean ± SEM. *P* values were determined by a two-tailed unpaired Students’ *t*-test in (**a**, **c**, **d**, **e**, **h**, **i**).

To understand the molecular mechanisms by which *Klf15* overexpression rescues the *Mrps5* loss of function phenotype in the heart, we conducted RNA sequencing to profile the transcriptomes of *Mrps5*^cKO^ mice infected with AAV9-*Klf15* or control AAV9-Luci. A total of 761 genes were significantly dysregulated, consisting of 421 genes that were upregulated and 340 genes that were downregulated (Fig. 7a, b).

The functional annotation of these differentially expressed genes was compared with our earlier analysis of the *Mrps5*^cKO^ mice and revealed some noteworthy features. For example, genes associated with the “oxidative phosphorylation” and “thermogenesis” pathways were significantly downregulated in both the uninjected and AAV-Klf15 injected *Mrps5*^cKO^ hearts (Figs. 3d, f, 7c), indicating that Klf15 overexpression did not restore the regulation of these pathways in *Mrps5*^cKO^ hearts. Meanwhile, the “glycolysis/gluconeogenesis” pathway genes are highly upregulated in AAV-*Klf15* injected *Mrps5*^cKO^ hearts, suggesting that *Klf15* overexpression induced a switch in the metabolic profile from oxidative phosphorylation to glycolysis (Fig. 7d). Additional analyses supported a role for expression of *Klf15* in *Mrps5*^cKO^ hearts in cardiac metabolism, endoplasmic reticulum stress and the unfolded protein response, as well as cardiac remodeling. Of particular importance was the restoration in the expression of genes related to “cardiac muscle contraction”, which was downregulated in the hearts of *Mrps5*^cKO^ mice but not in the hearts of AAV9-*Klf15* injected *Mrps5*^cKO^ mice. This observation is consistent with the findings showing *Klf15* overexpression rescued cardiac function in *Mrps5*^cKO^ mice.

We further investigated the expression profile for the “glucose metabolic biological processes” in *Mrps5*^cKO^ mice infected with AAV9-*Klf15* or AAV9-*Luci* by cross-referencing with the Gene Ontology (GO) database (Fig. 7e). These analyses revealed that many glucose metabolic biological processes were significantly enhanced in the hearts of *Mrsp5*^cKO^ mice upon *Klf15* re-expression. Conversely, we observed a significant reduction of the glycolysis-associated genes and proteins in Mrps5^cKO^ hearts (Supplementary Fig. 7a, b). A heatmap of the expression of representative genes and gene set enrichment analysis (GSEA) of important linked pathways further confirmed that expression of *Klf15* in *Mrps5*^cKO^ hearts induced a metabolic shift; these observations illustrate a switch from oxidative phosphorylation to glycolysis/gluconeogenesis (Fig. 7f–h). As a specific example, aldolase fructose-bisphosphate B (*Aldob*), a glycolytic enzyme that catalyzes the conversion of fructose-1,6-bisphosphate to glyceraldehyde 3-phosphate and dihydroxyacetone phosphate, was restored to normal levels in the hearts of AAV9-*Klf15* infected *Mrps5*^cKO^ mice (Fig. 7i, j). In addition, *Klf15* also rescues the decreased BCAAs catabolism in *Mrps5*^cKO^ hearts (Fig. 7f, h and Supplementary Fig. 8a–c). Overall, these data support a model in which *Klf15* overexpression promotes a switch to glycolysis and gluconeogenesis for energy production to compensate for impaired oxidative phosphorylation and enhanced BCAAs catabolism in the hearts of *Mrps5*^cKO^ mice.

### The mitochondrial metabolite l-phenylalanine and transcription factor c-myc mediate mitonuclear communication to repress *Klf15*

**Fig. 8:**
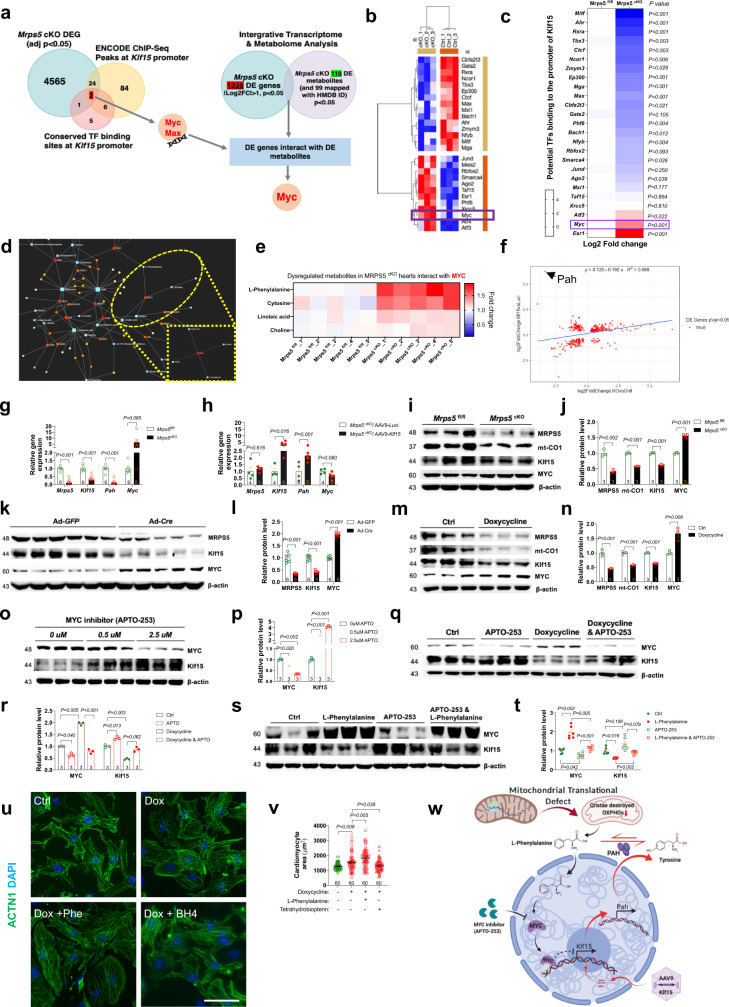
The metabolite l-phenylalanine and transcription factor myc mediate mitonuclear communication to repress KLF15. **a** Rationale used to identify potential upstream signals regulating Klf15 expression in *Mrps5*^cKO^ hearts. **b** Correlation of the heatmap and **c** relative expression of the candidate genes from the *Mrps5*^cKO^ RNA-sequencing data correlated with the ENCODE ChIP-Seq peaks over the *Klf15* gene promoter and/or the conserved transcription factor binding sites over the Klf15 promoter. **d** Dysregulated gene and metabolite interaction networks after *Mrps5* deletion. **e** Heatmap of the DE metabolites that interact with myc after *Mrps5* deletion. **f** Scatter diagram of dysregulated genes in *Mrps5*^cKO^ versus *Mrps5*^fl/fl^ hearts and *Mrps5*^cKO^/AAV-*Klf15* versus *Mrps5*^cKO^/AAV-*Luci* hearts highlighting Pah expression. The gene expression level of *Pah* is decreased in *Mrps5*^cKO^ hearts while it is elevated in *Mrps5*^cKO^/AAV-*Klf15* hearts. **g** qRT-PCR analysis of the expression level of *Mrps5*, *Klf15*, *Pah*, and *Myc* in *Mrps5*^fl/fl^ and *Mrps5*^cKO^ hearts and **h** *Mrps5*^cKO^/AAV-*Luci* and *Mrps5*^cKO^/AAV-*Klf15* hearts. **i**, **j** Representative immunoblot and quantification of protein levels of MRPS5, mt-CO1, KLF15, MYC, and ACTIN in the whole heart lysates of *Mrps5*^fl/fl^ and *Mrps5*^cKO^ mice. **k**, **l** Representative immunoblot and quantification of the protein level of MRPS5, KLF15, MYC, and ACTIN in whole cell lysates derived from NMCM cells from *Mrps5*^fl/fl^ mice after adenoviral Cre/GFP treatment for 5 days and **m**, **n** H9C2 cells after doxycycline treatment for 4 days (**m**, **n** also includes analysis of mt-CO1). **o**, **p** Representative immunoblot and quantification of protein levels of MYC and KLF15 in whole cell lysates from H9C2 cells after treatment with the MYC inhibitor APTO-253, **q**, **r** APTO-253 and/or doxycycline or **s**, **t** APTO-253 and/or l-Phenylalanine. **u**, **v** Representative images and cell size quantification of NMCMs treated with doxycycline and/or phenylalanine, tetrahydrobiopterin (BH4). **w** Schematic depiction of l-phenylalanine/MYC signaling axis demonstrating mechanism for repression of Klf15 expression generated by a defect in mitochondrial translation. Created with BioRender.com. N numbers are indicated in each panel. All data were presented as mean ± SEM. *P* values were determined by two-tailed unpaired Students’ *t*-test in (**a**, **c**, **g**, **h**, **j**, **l**, **n**). *P* values were determined by one-way ANOVA with Brown–Forsythe and Welch multiple comparisons test in (Fig. 8p, r, t, v).

We hypothesize that mitochondrial stresses and defects in mitochondrial protein translation resulting from the loss of *Mrps5* trigger the transcriptional repression of *Klf15*; this highlights the delicate balance, preserved through mitonuclear communication, which is required for the maintenance of the normal metabolic profile. We hypothesized that the decrease in *Klf15* expression in *Mrsp5*^cKO^ hearts was a result of transcriptional repression. We analyzed the *Mrps5*^cKO^ RNA-sequencing data in the context of the ENCODE ChIP-Sequence Peaks and conserved transcription factor binding sites over the *Klf15* promoter; this approach identified eight transcriptional regulators that putatively regulate *Klf15* (Supplementary Table 2). Among them, c-Myc and Max are differentially expressed in *Mrsp5*^cKO^ hearts (Fig. 8a–c), suggesting that they could participate in the regulation of the *Klf15* gene.

Next, we performed untargeted metabolome screening to determine the metabolite changes in *Mrsp5*^cKO^ hearts and integrated this information with the previously described expression dataset to identify the upstream metabolites (Fig. 8a–c). Both the volcano plot and the heatmap of dysregulated metabolites in *Mrps5*^cKO^ hearts showed a very tight grouping of the biological replicates (Supplementary Fig. 9a–d, *n* = 5). Approximately 70 metabolites were annotated as significantly changed. Examination of differentially expressed metabolites and pathway enrichment analysis revealed that “ABC transporters”, “protein digestion and absorption”, “aminoacyl-tRNA biosynthesis”, and “mineral absorption” pathways were significantly increased; in contrast, “purine metabolism”, “cGMP-PKG signaling pathway”, “renin secretion”, “Foxo signaling pathway” and “regulation of lipolysis in adipocytes” pathways were markedly reduced in *Mrsp5*^cKO^ hearts (Supplementary Fig. 9e, f). Further analysis of these top dysregulated pathways identified l-leucine, l-isoleucine, l-glutamine, and l-phenylalanine as the most commonly upregulated metabolites in the four most upregulated pathways (Supplementary Fig. 9g). Conversely, adenosine monophosphate (AMP) is the only downregulated metabolite commonly involved in the five most downregulated pathways (Supplementary Fig. 9h). These observations support a role for these metabolites in the defects identified in *Mrps5*^cKO^ hearts.

To better understand how dysregulated metabolites regulate *Klf15*, we performed integrative transcriptome and metabolome analysis using MetaboAnalyst. These analyses revealed that “aminoacyl-tRNA biosynthesis” and “protein digestion and absorption” are among the most dysregulated transcriptome and metabolome pathways in *Mrsp5*^cKO^ hearts (Supplementary Table 3). This is not surprising, given that loss of *Mrps5* led to mitochondrial protein translation defects. Interestingly, c-myc, which is a potential regulator of *Klf15* and differentially expressed in *Mrsp5*^cKO^ hearts, is associated with several metabolites, including L-phenylalanine (Fig. 8d, e and Supplementary Fig. 10), suggesting that they may mediate mitonuclear communication to repress *Klf15*. Intriguingly, transcriptome analysis also revealed that loss of *Mrps5* results in a dramatic increase in the expression of phenylalanine hydroxylase (PAH), which encodes an enzyme that catalyzes the hydroxylation of the aromatic side-chain of phenylalanine to generate tyrosine (Fig. 8f). This results in decreased levels of L-phenylalanine in *Mrsp5*^cKO^ hearts and generates a negative feedback loop that attempts to hold the system in check; overexpression of *Klf15* also increased the expression of PAH, disrupting the balance of l-phenylalanine (Fig. 8g, h).

We examined the protein levels of c-myc and KLF15 in the hearts of *Mrps5* cardiac knockout mice and found that c-myc expression was increased while KLF15 was decreased (Fig. 8i, j). Similarly, increased c-myc and decreased KLF15 levels were found in cardiomyocytes derived from the hearts of Ad-Cre-treated *Mrps5*^fl/fl^ neonatal mice compared with those treated with control Ad-GFP (Fig. 8k, l). Doxycycline (Dox) treatment has been shown to induce mitonuclear protein imbalance and extend longevity by inhibiting mitochondrial protein translation in mice^10,11^. Therefore, we treated cardiomyocytes with Dox and found that the expression of both MRPS5 and KLF15, together with that of mtDNA-encoded oxidative phosphorylation proteins (MTCO1), was reduced (Fig. 8m, n). In contrast, Dox treatment increased protein levels of c-myc (Fig. 8m, n), suggesting that c-myc is likely a downstream mediator of Dox-induced stress in cardiomyocytes. Next, we directly treated cardiomyocytes with APTO-253, a widely used myc inhibitor, and found that inhibition of c-myc resulted in increased expression of KLF15 (Fig. 8o, p). Furthermore, myc inhibitor treatment overrides the Dox-treatment c-myc protein level induction and restored KLF15 protein levels (Fig. 8q, r). Finally, we treated cells with l-phenylalanine and found that this produced an increase in the expression of c-myc, resulting in a decrease of KLF15 (Fig. 8s, t). As expected, chemical inhibition of c-myc expression could reduce c-myc protein levels and partially restore KLF15 levels reduced upon l-phenylalanine treatment (Fig. 8s, t).

In support of our hypothesis, a recent study demonstrated that dysregulated l-phenylalanine catabolism also played a key role in the processing of cardiac aging; pharmacological restoration of phenylalanine catabolism with tetrahydrobiopterin (BH4) could reverse age-associated cardiac impairment^17^. Given that loss of Mrps5 resulted in cardiac hypertrophy and increased L-phenylalanine, we tested whether tetrahydrobiopterin (BH4) treatment could suppress hypertrophy in cardiomyocytes. Indeed, L-phenylalanine treatment furtherly increased neonatal mouse cardiomyocyte size induced by doxycycline treatment. BH4 treatment suppressed doxycycline-induced cardiomyocyte hypertrophy (Fig. 8u, v). Therefore, these observations serve as an important indication of the therapeutic potential of augmenting l-phenylalanine catabolism to treat mitochondrial translation defect-associated cardiac diseases. Together, we propose a mechanism for L-phenylalanine-mediated mitonuclear communication in which c-myc protein levels are enhanced by this metabolite, resulting in the transcriptional repression of *Klf15* in the heart (Fig. 8w).

In addition to the l-phenylalanine/*c-myc* signaling axis, phosphorylation of CREB had also been identified as an upstream signal in the regulation of *Klf15*^18^. When we analyzed the gene subset of ENCODE ChIP-Seq peaks and conserved transcription factor binding sites in the *Klf15* promoter, we found sites for eight transcription factors (c-m*yc*, *Creb1*, *Max*, *Arnt*, *Nr3c1*, *Gata1*, *Usf1*, and *Usf2*). When compared with the DE metabolites identified in the hearts of *Mrps5*^cKO^ mice, only *Myc* and *Creb1* were present in both groups (Supplementary Fig. 11a). Indeed, the protein levels of phosphorylated CREB (p-CREB), but not that of total CREB is substantially lower in *Mrps5*^cKO^ hearts (Supplementary Fig. 11b, c). Consistent with a prior report, we found that the protein level for branched-chain amino acid transaminase 2 (Bcat2) is also downregulated in *Mrps5*^cKO^ hearts (Supplementary Fig. 11b, c). These findings suggest that the p-CREB/CREB signaling is involved in the regulation of *Klf15* upon *Mrps5* deletion. The DE metabolites that interact with Creb1 include AMP (adenosine monophosphate, AMP), adenosine, ADP (adenosine 5′-diphosphate, ADP), l-glutamine, and l-glutamic acid. Of these, AMP decreased most dramatically in *Mrps5*^cKO^ hearts (Supplementary Figs. 11d, e, 12). Both p-CREB and KLF15 were downregulated in doxycycline-stimulated cardiomyocytes as well (Supplementary Fig. 11f). Furthermore, AMP supplementation restored KLF15 expression, as well as that of p-CREB (Supplementary Fig. 11f). Taken together, our results demonstrate that both l-phenylalanine/MYC and AMP/p-CREB are key signaling nodes in the response of *Klf15* to mitochondrial translational stress (Supplementary Fig. 11g).

## Discussion

In this study, we report that *Mrps5* plays a vital role in maintaining normal mitochondrial function in the heart. We show that cardiac-specific loss of *Mrps5* in adult mouse causes severe pathological cardiac hypertrophy and heart failure, and cardiac-specific loss of *Mrps5* in mouse embryos causes abnormal heart development and embryonic lethality. Loss of function studies focused on other mitochondrial ribosomal proteins will be important to verify the generality and specificity of our data on mitochondrial ribosomal translation. In this study, we identified *Klf15* as an *Mrps5* target and demonstrated that *Klf15* restoration is able to rescue the cardiac defects in Mrps5 mutant mice. Our study reveals a mitonuclear communication axis mediated by *Mrps5* and *Klf15* in cardiomyocytes that signals the OXPHOS and glycolysis metabolism programs in response to mitochondrial stress. The identification of *Mrps5* as a marker of pathological cardiac hypertrophy provides important insights into the links between the mitochondrial ribosomal translational machinery and normal cardiac function. Our studies further indicate that metabolic reprogramming by increasing glycolysis/gluconeogenesis in response to decreased OXPHOS could promote cell survival and benefit failing hearts.

Pathological cardiac hypertrophy is a complex biological process, which involves both transcriptional and post-transcriptional regulation of cardiac gene expression. In the hearts of *Mrps5*^cKO^ mice, cardiac hypertrophy manifested before we observed any defects in mitochondrial function. The cardiac hypertrophy rapidly progressed to heart failure once disruption of mitochondrial structure and function became detectable. Analyses of both the transcriptome and metabolome confirmed the identity of dysregulated genes in *Mrps5*^cKO^ hearts; these genes included many that are involved in the development of cardiac hypertrophy and cardiomyocyte contraction. As expected, pathways related to OXPHOS and metabolism were also linked to the onset of cardiac defects in *Mrps5*^cKO^ hearts. We propose that loss of *Mrps5* in the heart impairs mitochondrial protein translation. This produces an imbalanced metabolism that leads to the initial cardiac hypertrophy and the eventual heart failure observed in these animals.

KLF15 is a well-characterized transcription factor, and previous studies have demonstrated that KLF15 regulates cell differentiation^19,20^, circadian rhythm^21,22^, and cellular metabolism^23^. *Klf15* deficiency occurs in human cardiomyopathy and aortic aneurysms, and its deletion leads to cardiomyopathy and aortopathy in mice^24^. KLF15 was shown to repress cardiac hypertrophy, while *Klf15* knockout mice develop cardiac hypertrophy in response to pressure overload^25^. We described a decrease in *Klf15* expression in response to the loss of *Mrps5* in the heart. Therefore, we propose that loss of *Klf15* mediates the development of cardiac hypertrophy and heart failure in *Mrps5*^cKO^ hearts in response to mitochondrial stress. Interestingly, KLF15 strongly promotes metabolic reprogramming in the *Mrps5* null heart even after the collapse of most mitochondrial cristae; there is an increase in glycolysis and a decrease in mitochondrial OXPHOS. While studies show preservation of fatty acids utilization is cardioprotective^26,27^, others show enhanced glucose utilization is a compensatory process, which may be beneficial to the stressed hearts^28–30^. In addition to its function in regulating cardiac hypertrophy, previous research also demonstrated that *Klf15* plays an important role in the regulation of gluconeogenesis^31^. Our results revealed that the observed metabolic reprogramming (enhanced glycolysis and reduced OXPHOS) substantially repressed cardiac hypertrophy and pathological remodeling of the heart. Our study provides further evidence of the fine balance that exists in the heart between the two major energy-producing systems and how they are tuned to ensure normal cardiac function under stress conditions. As a compensatory mechanism in response to mitochondrial stress, *Mrps5* mutant cardiomyocytes turn on the glycolysis pathway mediated by Klf15-dependent signaling. We also found that protein processing in the endoplasmic reticulum and the unfolded protein response (also known as a cytoprotective signaling pathway upon cellular stress) were enhanced after *Klf15* was reintroduced into *Mrps5*^cKO^ hearts. These results are consistent with prior findings that report an increase in the expression of genes involved in glycolysis during mitochondrial stress. As we have also proposed, these studies further support enhanced glycolysis/gluconeogenesis as a means to provide more energy to sustain normal cardiac function^32,33^.

Interestingly, the rescue experiments using the top candidate genes identified from both the transcriptome and proteome screening (*Adra1a*, *Angpt1*, *Ces1d*, *Enpp2*, *Klf15*, and *Pik3r1*) produced degrees of rescue in *Mrps5*^cKO^ mice, indicating a differential requirement for these molecular pathways in the maintenance of cardiomyocyte metabolism and cardiac function. Using echocardiography at set intervals, we demonstrate that cardiac function is essentially preserved during the first 10 weeks post *Mrps5* deletion. Intervention during this period using cardiac expression of the six prioritized genes identified as downregulated in the *Mrps5*^cKO^ mouse hearts demonstrated rescue of the later-stage heart phenotype for all treatments except *Pik3r1* (the one gene whose expression in DCM patients was not consistent with our observations).

There is then a transition between 10–12 weeks post *Mrps5* deletion in which the mitochondrial cristae start to collapse and mitochondrial function becomes impaired; the data illustrates that *Klf15* and *Adra1a* still maintain an ability to rescue the cardiac phenotype. However, at later stages following *Mrps5* ablation (after 12 weeks post *Mrps5* deletion), mitochondrial cristae have largely collapsed, resulting in the loss of essentially all mitochondrial function in cardiomyocytes; while expression of *Adra1a* is no longer able to rescue the heart defects at these late stages, *Klf15* expression in *Mrps5*^cKO^ hearts still demonstrates limited improvement in heart function. Interestingly, the loss in *Adra1a* effectiveness coincides with the stage at which we observe the destruction of the mitochondrial cristae; after the mitochondrial cristae were destroyed, mitochondrial ribosomal translation stalled, and almost all mitochondrial function was lost. This loss of mitochondrial function would result in a decrease in the energy pool required for cardiac function and this would constrain the ability of *Adra1a* to enhance cardiac contractility as previously described in refs. ^34,35^. Therefore, we propose that some mitochondrial function is required for *Adra1a* treatment to be effective in the *Mrps5*^cKO^ heart. Consistent with previous reports, we observed that re-expression of *Adra1a* in *Mrps5*^cKO^ hearts did not induce cardiac hypertrophy.

We propose that *Klf15* rescues the *Mrps5*^cKO^ cardiac defects by distinct mechanisms before and after the collapse of mitochondrial cristae and loss of mitochondrial function. In the early stages during which mitochondrial function is retained, the primary role of Klf15 is to inhibit cardiac hypertrophy by regulating hypertrophic gene expression. Once mitochondrial function becomes severely impaired as a result of *Mrps5* deletion, *Klf15* is able to mediate an enhancement of the glycolysis pathway to supplement the energy demands of the *Mrps5*^cKO^ hearts and enable the animals to survive. The twofold effect of this later Klf15 action is to enhance energy production by glycolysis and gluconeogenesis and reduce the dependence on mitochondrial OXPHOS; the result is a slowing of the deterioration of the *Mrps5*^cKO^ hearts and preservation of cardiac function.

*Klf15* has also been shown to be a key regulator of BCAAs catabolism; it is sharply decreased upon glucose stimulation which downregulates *Bcat2*, resulting in the accumulation of BCAAs. This process also activates mTOR signaling and metabolic reprogramming, which occurs during cardiomyocyte hypertrophy and heart failure^18,36^. Additionally, high concentrations of BCAAs have been shown to suppress the expression of KLF15. Our transcriptomic profiling of *Mrps5*^cKO^ hearts showed that BCAAs degradation-associated signaling pathways were significantly reduced. After *Klf15* re-expression in *Mrps5*^cKO^ hearts, we found that expression of BCAA catabolism-associated genes was enhanced via the AMP-CREB pathway, consistent with a previous report^18^. These findings suggest that the *Klf15-*BCAAs catabolism-metabolism reprogramming may also play an important role in rescuing the *Mrps5* null hearts.

The purpose of mitonuclear communication (both anterograde, from nucleus to mitochondria, and retrograde, from mitochondria to nucleus) is to maintain homeostasis of a cell both under basal conditions and in response to a variety of stresses. Mutations in genes involved in mitonuclear communication, as well as imbalanced signals derived from impaired mitonuclear responses, are often linked to developmental defects and human diseases. Our study provides important insights into this poorly characterized communication nexus, particularly in the retrograde direction. We provide evidence for Klf15 as an essential mediator of mitonuclear communication in response to a loss of Mrps5 in the heart. We further describe how Klf15 is able to relay signals via l-phenylalanine/c-myc and AMP/p-CREB from mitochondria to nuclei to activate a gene expression program that is able to modulate cardiac metabolism and hypertrophy. In conclusion, our data shed light on how mitochondrial ribosomal translational defects in mammalian cardiomyocytes are able to have profound biological implications for cardiac function. These observations also support a role for metabolic reprogramming as a potential strategy for reducing cardiac hypertrophy and pathological remodeling driven by defects in mitochondrial translation.

## Methods

Data, analytic methods, and study materials will be made available to other researchers for purposes of reproducing the results or replicating procedures, on request, by direct communication. Analytic assays on tissue samples were performed by laboratory staff in a blinded fashion. A detailed description of materials and methods is available in the Supplementary Information.

### Human tissue sampling study protocol

Left ventricular (LV) tissues were taken from patients with terminal-stage heart failure indicated for heart transplantation. In brief, the patient’s heart was removed at the time of transplantation, and LV tissue was subsequently dissected and snap-frozen. We used LV samples from healthy hearts that were not implanted to serve as controls. All experimental protocols involving patients were approved by the Ethics Committee of the Second Affiliated Hospital Zhejiang University School of Medicine.

### Animal studies

All protocols concerning animal studies were approved by the Institutional Animal Care and Use Committees at Zhejiang University, Boston Children’s Hospital, and the University of South Florida. The human study protocol using heart tissue from DCM patients and control patients was approved by the Ethics Committee of the Second Affiliated Hospital of Zhejiang University. Patients provided written informed consent.

### AAV9 preparation and injection

The cDNA fragments encoding *Luciferas*e, *Gfp*, *Klf15*, *Adra1a*, *Angpt1*, *Ces1d*, *Enpp2*, or *Pik3r1* were separately cloned into the ITR (inverted terminal repeats)-containing AAV9 plasmid harboring the chicken cardiac TNT promoter, to yield AAV9-*cTnT*-*Luciferase* (AAV9-*Luci*), AAV9-*cTnT*-*Gfp* (AAV9-*Gfp*), AAV9-*cTnT*-*Klf15* (AAV9-*Klf15*), AAV9-*cTnT*-*Adra1a* (AAV9-*Adra1a*), AAV9-*cTnT*-*Angpt1* (AAV9-*Angpt1*), AAV9-*cTnT*-*Ces1d* (AAV9-*Ces1d*), AAV9-*cTnT*-*Enpp2* (AAV9-*Enpp2*), and AAV9-*cTnT*-*Pik3r1* (AAV9-*Pik3r1*). AAV9 was packaged in 293 T cells with AAV9: Rep-Cap and pHelper (pAd deltaF6, Penn Vector Core) and purified and concentrated by gradient centrifugation. AAV9 titer was determined by quantitative PCR. Adult mice were treated with AAV9 1–2 × 10^12^ particles/heart, adult mice were anesthetized, and thoracotomy was performed through the fourth intercostal space. The ascending aortic artery and the main pulmonary artery were clamped. The AAV9 was injected at a volume of 100 µl through the tip of the heart into the left ventricular cavity. The arteries were occluded for 10 s after the AAV9 injection. Neonatal mice (postnatal day 1) were treated with AAV9 2–5 × 10 particles/pup by subcutaneous injection according to our prior reports^37^. These mice were then treated with tamoxifen at the adult stage (4 weeks old for 1-week treatment) to induce KO of *Mrps5*. Hearts were then collected 14 weeks post tamoxifen injection (at 19 weeks of age).

### Mouse models of pathological hypertrophy/cardiac disease

To generate a mouse model of pathological cardiac hypertrophy/disease, transverse aortic constriction (TAC) surgery was performed on 8–10-week-old mice. In brief, mice were anesthetized with 4% chloral hydrate. Then the transverse aorta was constricted against a 27-gauge needle. The sham group animals underwent mock surgery without aortic constriction.

Myocardial infarction (MI) surgery was performed on 8–10-week-old mice by permanent ligation of the left anterior descending (LAD) branch of the coronary artery. In brief, mice were anesthetized with 4% chloral hydrate and the chest was shaved and cleaned with alcohol. Ventilation was performed with a tidal volume of 225 µl for a 25 g mouse and a respiratory rate of 130 breaths per minute. 100% oxygen was provided to the inflow of the ventilator. The chest was opened through a left parasternal incision, and the heart was exposed at the left 3rd–4th intercostal space (a chest retractor was applied to facilitate access). The pericardium was opened, and the LAD coronary artery was ligated using 8-0 silk sutures. The lungs were slightly overinflated to assist in the removal of air from the pleural cavity. The dissected intercostal space and chest skin were closed using a 6-0 silk suture.

### In vivo echocardiography

Echocardiographic measurements were performed on *Mrps5*^fl/fl^ and *Mrps5*^cKO^ mice using a Visual Sonics Vevo 2100 Imaging System (Visual Sonics, Toronto, Canada) with a 40 MHz MicroScan transducer. Heart rate and LV dimensions, including diastolic and systolic wall thicknesses, LV end-diastolic and end-systolic chamber dimensions, were measured from the 2D short-axis under M-mode tracings at the level of the papillary muscle. LV mass and functional parameters such as percentage of fractional shortening (FS %) and left ventricular volume were calculated using the above primary measurements and accompanying software.

### Neonatal cardiomyocyte isolation and culture

Hearts from postnatal day 1 C57BL/6 J pups were harvested and washed with PBS to remove blood cells. Ventricular tissue was incubated with enzymes and buffers supplied by the Miltenyi neonatal mice cardiomyocyte isolation kit as indicated. Then cardiac tissue was digested and triturated by pipetting and filtered through a 70 µm cell strainer. NMCMs were then collected by centrifuging at 1000 rpm for 5 min and resuspended in DMEM medium supplemented with 10% fetal bovine serum (FBS) and 1% penicillin/streptomycin. Cells were allowed to attach for 20 min, then NMCMs were collected and maintained in DMEM containing 10% FBS and 1% penicillin/streptomycin. NMCMs were deprived of serum for 24 h prior to neurohumoral treatment to induce cardiomyocyte hypertrophy.

### Quantification of cell size

In vitro: Neonatal cardiomyocytes were stimulated with PE/ISO/FBS for 48 h, stained with ACTN1 and DAPI. Immunofluorescence images were taken with a Nikon N1 microscope, cell size was quantified using Image J software. In vivo: Heart sample slides were stained with Wheat Germ Agglutinin (WGA), ACTN1, and DAPI; fluorescence images were taken with a Nikon N1 microscope, and cell size were quantified via Image J software.

### Quantification of cardiac fibrosis

Heart sample slides were stained with Sirius Red/Fast Green. In brief, slides were stained with Sirius Red for 2 h and Fast Green for 15 min; bright field images of the slides were taken, and areas of fibrosis were quantified with Image J software.

### Measurement of ATP content

An ATP assay kit (Beyotime, cat #S0026) was used to determine the ATP content of mouse hearts. Tissue from fresh mouse heart tissue blocks was lysed with 100 µl lysis buffer ATP lysis buffer per 20 mg heart tissue with thorough grinding (IKA, T10 basic ULTRA-TURRAX). The tissue was then centrifuged at 12,000 × *g* at 4 °C for 5 min. The sample supernatant was transferred to a new tube for ATP detection. ATP standard solutions were prepared at 7 micromolar concentrations (0.01, 0.03, 0.1, 0.3, 1, 3, and 10). ATP assay buffer was diluted with ATP dilution buffer at a ratio of 1:9 to prepare the ATP assay working buffer. About 100 µL ATP assay working buffer was added to each well of a 96-well plate and allowed to stand for 5 min, then 20 µL of sample supernatant or ATP standard solution was added. RUM value was detected using a Molecular Devices, SpectraMax M5 multi-detection microplate reader system. The ATP relative count content was also determined according to the ATP standard solution RUM value.

### Measurement of cardiac muscle fiber oxygen consumption rate (OCR)

The OCR of cardiac muscle fibers were detected with Oxygraphy-2k from OROBOROs instruments using standard protocols^38,39^. In brief, heart muscle tissue was cut into small samples of 10–20 mg and put into a 50 ml falcon tube with 10 ml of ice-cold BIOPS. Fiber bundles were then separated mechanically with two very sharp forceps in a small petri dish, on ice. The degree of separation was evaluated by observing a change from red to pink in the separated fiber bundles. After tissue separation, the fiber bundles were placed sequentially into 2 mL of ice-cold BIOPS containing 20 µL of saponin stock solution (5 mg/mL; final concentration 50 µg/mL) into individual wells of a falcon 12-well tissue culture plate, and shaken using gentle agitation on ice for 30 min. Then all samples were quickly transferred from the saponin solution into 2 ml of MiR06 (Mitochondrial respiration medium) buffer and shaken further with gentle agitation for 10 min on ice. Weight measurements were made after permeabilization and before adding the tissue into the O2k chamber; samples were selected in size to comprise several loosely connected fiber bundles of 0.5–2 mg wet weight. The bundles were carefully blotted on filter paper (samples were taken using sharp forceps (angular tip) and placed onto the filter paper). Immediately after reading the wet weight, the sample was transferred into a well with 2 ml ice-cold MiR05 buffer. At the same time, baseline respiration was measured (at 37 °C for mammalian tissues) in MiR06 designed for optimal protection of mitochondrial function, then the fiber bundles were put into each chamber. Oxygen was allowed to flow into each chamber and then closed. Once the respiration curves stabilized, substrates and inhibitors of each electron transfer chain (ETC) complex were put into the chambers in order. Pyruvate, malate, glutamate, and ADP as the substrates of complex I; Rotenone as the inhibitor of complex I; Succinate as the substrate of complex II, antimycin A as the inhibitor of complex III; Ascorbate and TMPD as the substrates of complex IV, and sodium azide as the inhibitor of complex IV.

### Quantification of mitochondrial cristae length

Murine heart tissue or NMCMs were fixed immediately after harvest using 2.5% glutaraldehyde. Mitochondrial morphology was observed using transmission electron microscopy, and images of mitochondria were then analyzed with Image J software; the mitochondrial cristae length and mitochondrial area were further quantified from these images.

### Isolation of mitochondria from heart tissue

Mitochondria were isolated from murine heart tissue as previously described with some modifications^40^. In brief, mice were euthanized by decapitation and heart tissue was immediately washed in ice-cold IB-1 buffer (three to four times with ice-cold IB-3 to remove the blood), then tissue was cut into small pieces using scissors. The used IB-3 solution was discarded and the tissue was washed once again with 10 ml of fresh, ice-cold IB-1. The heart tissue was transferred to an ice-cold glass/Teflon Potter Elvehjem homogenizer. IB-1 was added in the ratio of 4 ml of buffer per gram of heart tissue. Homogenization, as well as the following steps, were carried out at 4 °C to minimize the activation of proteases and phospholipases. After homogenization was completed, tissue was transferred to a centrifuge and spun at 740 × *g* for 5 min at 4 °C (repeated 2–3 times). The supernatant was collected and centrifuged at 9000 g for 10 min at 4 °C. The supernatant (representing the cytosolic fraction containing lysosomes and microsomes) was then discarded, and the pellet (containing the mitochondria) was gently resuspended in 20 ml of ice-cold IB-2. The mitochondrial suspension was then centrifuged at 10,000 × *g* for 10 min at 4 °C (repeated two times). The pellet representing the crude mitochondrial fraction was used for further experimentation.

### RNA sequencing

Cardiac tissues were harvested from 12-week-old littermate control *Mrps5*^fl/fl^ and *Mrps5*^cKO^ mice. Ages and genotypes of AAV9 injected mice were recorded. RNA-seq experiments were performed by Novogene (Beijing, China). Briefly, total RNA was isolated from fresh ventricular tissue using TRIzol (Invitrogen). RNA integrity was assessed using the RNA Nano 6000 Assay Kit of the Bioanalyzer 2100 system (Agilent Technologies, CA, USA). A total amount of 1 μg RNA per sample was used as input material for the RNA sample preparations. Briefly, mRNA was purified from total RNA using poly-T oligo-attached magnetic beads. Fragmentation was carried out using divalent cations under elevated temperature in First-Strand Synthesis Reaction Buffer (5X). First-strand cDNA was synthesized using a random hexamer primer and M-MuLV Reverse Transcriptase (RNase H-). Second-strand cDNA synthesis was subsequently performed using DNA Polymerase I and RNase H. Remaining overhangs were converted into blunt ends via exonuclease/polymerase activities. After polyadenylation of 3′ ends of DNA fragments, adapters with hairpin loop structures were ligated to prepare for hybridization. In order to select cDNA fragments of the preferred length (~370–420 bp), the library fragments were purified with the AMPure XP system (Beckman Coulter, Beverly, USA). Then PCR was performed with Phusion High-Fidelity DNA polymerase, Universal PCR primers, and Index (X) Primer. PCR products were then purified (AMPure XP system) and library quality was assessed on the Agilent Bioanalyzer 2100 system. The clustering of the index-coded samples was performed on a cBot Cluster Generation System using TruSeq PE Cluster Kit v3-cBot-HS (Illumia) according to the manufacturer’s instructions. After cluster generation, the library preparations were sequenced on an Illumina Novaseq platform and 150 bp paired-end reads were generated. Raw data (raw reads) of fastq format were first processed through in-house perl scripts. In this step, clean data (clean reads) were obtained by removing reads containing adapter, reads containing ploy-N, and low-quality reads from raw data. At the same time, Q20, Q30, and GC content of the clean data were calculated. All the downstream analyses were based on clean data with high quality. Reference genome and gene model annotation files were downloaded from the genome website directly. The index of the reference genome was built using Hisat2 v2.0.5 and paired-end clean reads were aligned to the reference genome using Hisat2 v2.0.5. We selected Hisat2 as the mapping tool for that Hisat2 can generate a database of splice junctions based on the gene model annotation file and, thus, a better mapping result than other non-splice mapping tools. FeatureCounts v1.5.0-p3 was used to count the read numbers mapped to each gene. The FPKM of each gene was calculated based on the length of the gene and the reads count mapped to this gene. FPKM, the expected number of fragments per Kilobase of transcript sequence per millions of base pairs sequenced, considers the effect of sequencing depth and gene length for the reads count at the same time, and is currently the most commonly used method for estimating gene expression levels. Differential expression analysis of two conditions/groups (two biological replicates per condition) was performed using the DESeq2 R package (1.20.0). DESeq2 provides statistical routines for determining differential expression in digital gene expression data using a model based on the negative binomial distribution. The resulting *P* values were adjusted using Benjamini and Hochberg’s approach for controlling the false discovery rate. Genes with an adjusted *P* value <0.05 found by DESeq2 were assigned as differentially expressed. Gene Ontology (GO) enrichment analysis of differentially expressed genes was implemented by the ClusterProfiler R package, in which gene length bias was corrected. GO terms with corrected *P* value less than 0.05 were considered significantly enriched by differentially expressed genes. KEGG is a database resource for understanding high-level functions and utilities of the biological system, such as the cell, the organism, and the ecosystem, from molecular-level information, especially large-scale molecular datasets generated by genome sequencing and other high-throughput experimental technologies (http://www.genome.jp/kegg/). We used ClusterProfiler R package to test the statistical enrichment of differential expression genes in KEGG pathways.

### Metabolomics sequencing

Metabolomics sequencing and analyses were performed using an UHPLC (1290 Infinity LC, Agilent Technologies) coupled to a quadrupole time-of-flight (AB Sciex TripleTOF 6600) using services provided by Shanghai Applied Protein Technology. Hearts from 12-week-old *Mrps5*^fl/fl^ and *Mrps5*^cKO^ mice were collected in 5 mL vacutainer tubes containing the chelating agent ethylene diamine tetraacetic acid (EDTA), then samples were centrifuged for 15 min (1500 × *g*, 4 °C). Each aliquot (150 μL) of the plasma sample was stored at −80 °C until analysis by UPLC-Q-TOF/MS. The plasma samples were thawed at 4 °C and 100 μL aliquots were mixed with 400 μL of cold methanol/acetonitrile (1:1, v/v) to remove the protein. The mixture was centrifuged for 15 min (14,000 × *g*, 4 °C), then the supernatant was dried in a vacuum centrifuge. For LC-MS analysis, the samples were re-dissolved in 100 μL acetonitrile/water (1:1, v/v) solvent. To monitor instrument stability and repeatability, quality control (QC) samples were prepared by pooling 10 μL of each sample and analyzed together with the other samples. The QC samples were inserted regularly and analyzed every five samples. In both ESI positive and negative modes, the mobile phase contained A = 25 mM ammonium acetate and 25 mM ammonium hydroxide in water and B = acetonitrile. The gradient was 85% B for 1 min and was linearly reduced to 65% in 11 min. It was then reduced to 40% in 0.1 min and maintained for 4 min. It was then increased to 85% in 0.1 min, with a 5 min re-equilibration period. The ESI source conditions were set as follows: Ion Source Gas1 (Gas1) as 60, Ion Source Gas2 (Gas2) as 60, curtain gas (CUR) as 30, source temperature: 600°C, IonSpray Voltage Floating (ISVF) ± 5500 V. In MS-only acquisition, the instrument was set to acquire over the m/z range 60–1000 Da, and the accumulation time for TOF MS scan was set at 0.20 s/spectra. In auto MS/MS acquisition, the instrument was set to acquire over the m/z range 25–1000 Da, and the accumulation time for product ion scan was set at 0.05 s/spectra. The product ion scan is acquired using information-dependent acquisition (IDA) with high sensitivity mode selected. The parameters were set as follows: the collision energy (CE) was fixed at 35 V with ±15 eV; declustering potential (DP), 60 V (+) and −60 V (−); exclude isotopes within 4 Da, candidate ions to monitor per cycle: 10.

For data processing, the raw MS data (wiff.scan files) were converted to MzXML files using ProteoWizard MSConvert before importing into XCMS (freeware). For peak picking, the following parameters were used: centWave m/z = 25 ppm, peak width = c (10, 60), prefilter = c (10, 100). For peak grouping, parameters were set as follows: bw = 5, mzwid = 0.025, minfrac = 0.5. CAMERA (Collection of Algorithms of MEtabolite pRofile Annotation) was used for the annotation of isotopes and adducts. In the extracted ion features, only the variables having more than 50% of the nonzero measurement values in at least one group were kept. Compound identification of metabolites was performed by comparing the accuracy m/z value (<25 ppm) and MS/MS spectra with an in-house database established with available authenticated standards.

### Western blot

Protein lysate samples were prepared from cultured cells and heart tissues using cell and tissue extraction reagents (Invitrogen, FNN0011 and FNN0071) supplemented with proteinase inhibitors. Lysate samples (30 μg total protein for each) were separated by 6 or 10% SDS–PAGE and electrophoretically transferred to PVDF membranes. MRPS5 protein was detected with rabbit antibody to MRPS5 (Gene Tex, GTX103930; 1:1000 dilution). mt-ATP6 protein was detected with mouse antibody to mt-ATP6 (Abcam, ab219825; 1:1000 dilution). mt-CO1 protein was detected with rabbit antibody to mt-CO1 (Abcam, ab203912; 1:1000 dilution). mt-ND1 protein was detected with rabbit antibody to mt-ND1 (Abcam, ab181848; 1:1000 dilution). COX IV protein was detected with mouse antibody to COX IV (CST, 4844 S; 1:1000 dilution). VDAC protein was detected with rabbit antibody to VDAC (CST, 4661 S; 1:1000 dilution). p-CREB protein was detected with rabbit antibody to p-CREB (CST, 9198 S; 1:1000 dilution). CREB protein was detected with rabbit antibody to CREB (CST, 9197 S; 1:1000 dilution). KLF15 protein was detected with rabbit antibody to KLF15 (Abcam, ab2647; 1:1000 dilution). BCAT2 protein was detected with rabbit antibody to BCAT2 (Abcam, ab95976; 1:1000 dilution). MYC protein was detected with rabbit antibody to MYC (CST, 9402 S; 1:1000 dilution). ALDOB protein was detected with rabbit antibody to ALDOB (HUABIO, ER62642; 1:1000 dilution). HK1 protein was detected with rabbit antibody to HK1 (HUABIO, ST47-05; 1:1000 dilution). GLUT1 protein was detected with rabbit antibody to GLUT1 (HUABIO, ET1601-10; 1:1000 dilution). GLUT4 protein was detected with rabbit antibody to GLUT4 (HUABIO, R1402-3; 1:1000 dilution). GAPDH protein was detected with rabbit antibody to GAPDH (HUABIO, R1210-1; 1:5000 dilution). β-Actin protein was detected with mouse antibody to β-Actin (HUABIO, EM2001-07; 1:5000 dilution). a-tubulin protein was detected with a mouse antibody to a-tubulin (CST, 2144 S; 1:5000 dilution). Protein bands were visualized with the Bio-Rad ChemiDoc imaging system.

### Quantitative reverse transcription polymerase chain reaction (qRT-PCR)

Heart tissue samples or mice cardiomyocytes were homogenized, and then total RNA was extracted with Trizol reagent (Thermo Fisher Scientific) as per the manufacturer’s instructions. Reverse transcription was performed using the HiScript Reverse Transcriptase kit (Vazyme). qRT-PCR was performed using ChamQ SYBR Color qPCR Master Mix (Vazyme) according to the manufacturer’s protocol. qRT-PCR was performed on a Vii7 qPCR machine (Thermo Fisher Scientific) with cycle threshold (CT values) normalized to an endogenous control (18 S RNA or actin) and relative expression was calculated comparing the average change in CT between samples. PCR primers for qRT-PCR were provided in the associated figure legends.

### Statistical analysis

All data were presented as scatter dot plots or bar plots with mean ± standard error of mean (SEM), and *p* < 0.05 was considered significant. As to comparison between the two groups, a two-tailed unpaired Students’ *t*-test was used as indicated. For more than two groups, one-way ANOVA with the Brown–Forsythe and Welch multiple comparisons test was used. Statistical analysis and plotting were performed using GraphPad Prism 9.0 (GraphPad Software, San Diego, CA, USA).

### Reporting summary

Further information on research design is available in the Nature Portfolio Reporting Summary linked to this article.

## Supplementary information

Supplementary Information

Reporting Summary

## Data Availability

RNA-seq data has been deposited in GEO under accession codes: GSE200940, GSE200999, and GSE201022. All data and reagents described in this manuscript will be administered in accordance with both the University of South Florida (USF) and NIH policies, including the NIH Data Sharing Policy and Implementation Guidance of March 5, 2003. Source data are provided with this paper.

## Source data

Source Data
