# Supplementary material for: A defect in mitochondrial protein translation influences mitonuclear communication in the heart
Source: Nat Commun. 2023 Mar 22;14:1595. doi: 10.1038/s41467-023-37291-5 (PMC10033703; doi:10.1038/s41467-023-37291-5)
Supplement: Supplementary file 1 — Supplementary Information [file 41467_2023_37291_MOESM1_ESM.pdf]

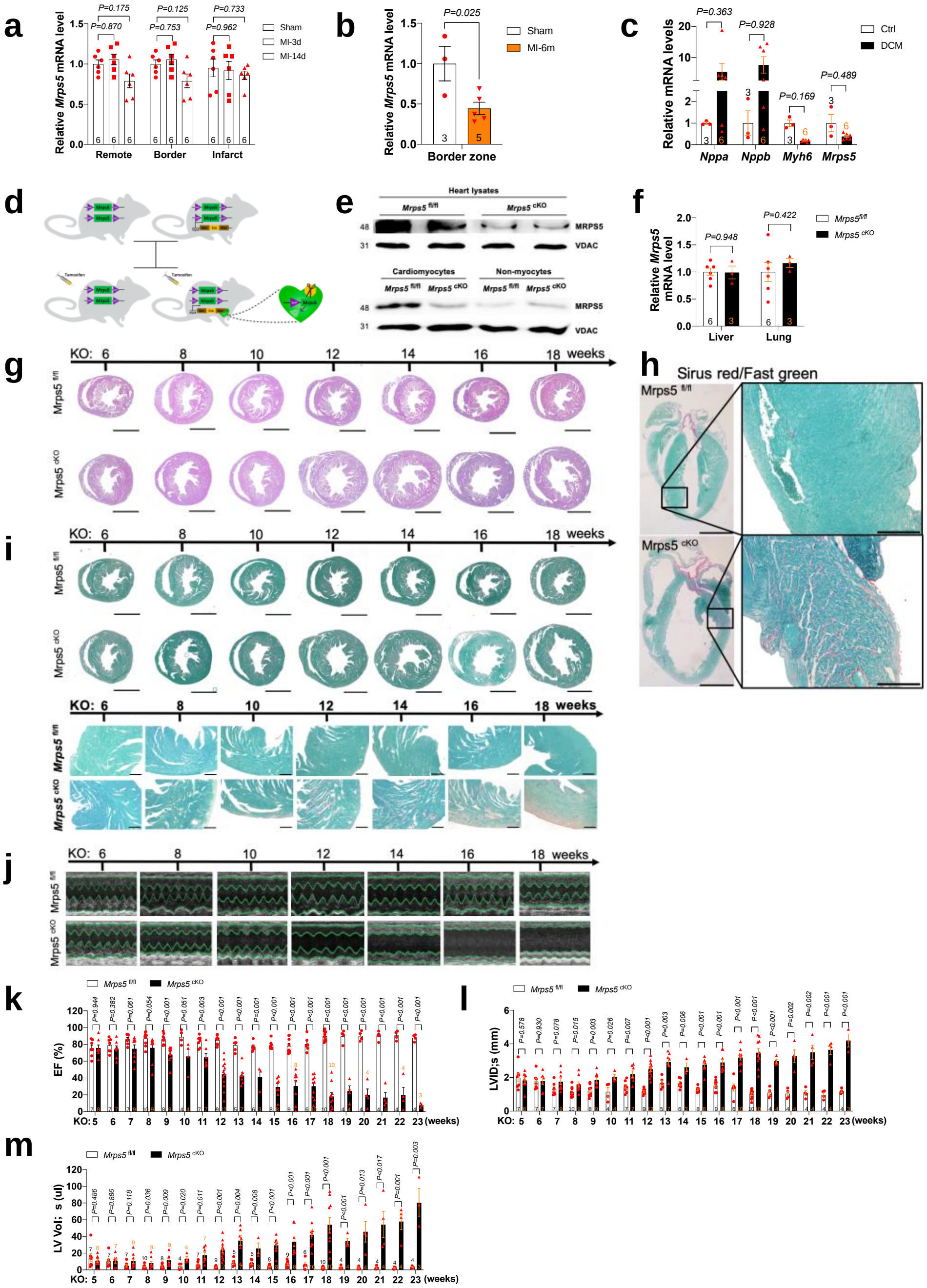

Supplementary Figure 1. Cardiomyocyte-specific *Mrps5* deletion results in cardiac defects. a. *Mrps5* expression in distinct regions (remote zone, border zone and infarct zone) of mouse hearts of sham or myocardial infarction (MI) groups at 3d and 14d post MI surgery. **b.** *Mrps5* expression in mouse hearts of sham or myocardial infarction (MI) groups 6m post MI surgery. **c.** RT-qPCR detecting expression of *Nppa*, *Nppb*, *Myh6* and *Mrps5* in human control or dilated cardiomyopathy (DCM) hearts. **d.** Methodology used for generation of cardiac specific, inducible *Mrps5* mutant mice. **e.** Representative immunoblot demonstrating MRPS5 protein levels in mice whole heart lysates, cardiomyocytes and non-myocytes derived from *Mrps5*$^{fl/fl}$ and *Mrps5*^{cKO} mouse hearts. **f.** Gene expression analysis of *Mrps5* from *Mrps5*$^{fl/fl}$ and *Mrps5*^{cKO} mouse liver and lung. **g.** Representative images of H&E stained cross sections from *Mrps5*$^{fl/fl}$ and *Mrps5*^{cKO} mouse hearts. Scale bar = 500 µm. **h.** Representative images of Sirus Red and Fast Green stained longitudinal sections from *Mrps5*$^{fl/fl}$ and *Mrps5*^{cKO} mouse hearts. Scale bar = 500 µm. **i.** Representative images of Sirus Red and Fast Green stained cross sections from *Mrps5*$^{fl/fl}$ and *Mrps5*^{cKO} mouse hearts. Scale bar = 500 µm. **j.** Representative examples of M-mode echocardiography from *Mrps5*$^{fl/fl}$ and *Mrps5*^{cKO} mice. **k-m.** Echocardiographic measurement of cardiac function in *Mrps5*$^{fl/fl}$ and *Mrps5*^{cKO} mice at 5 to 23 weeks after tamoxifen injection. N numbers are indicated in each panel. All data are presented as mean ± SEM. P values were determined by 2-tailed unpaired Students' *t*-test.

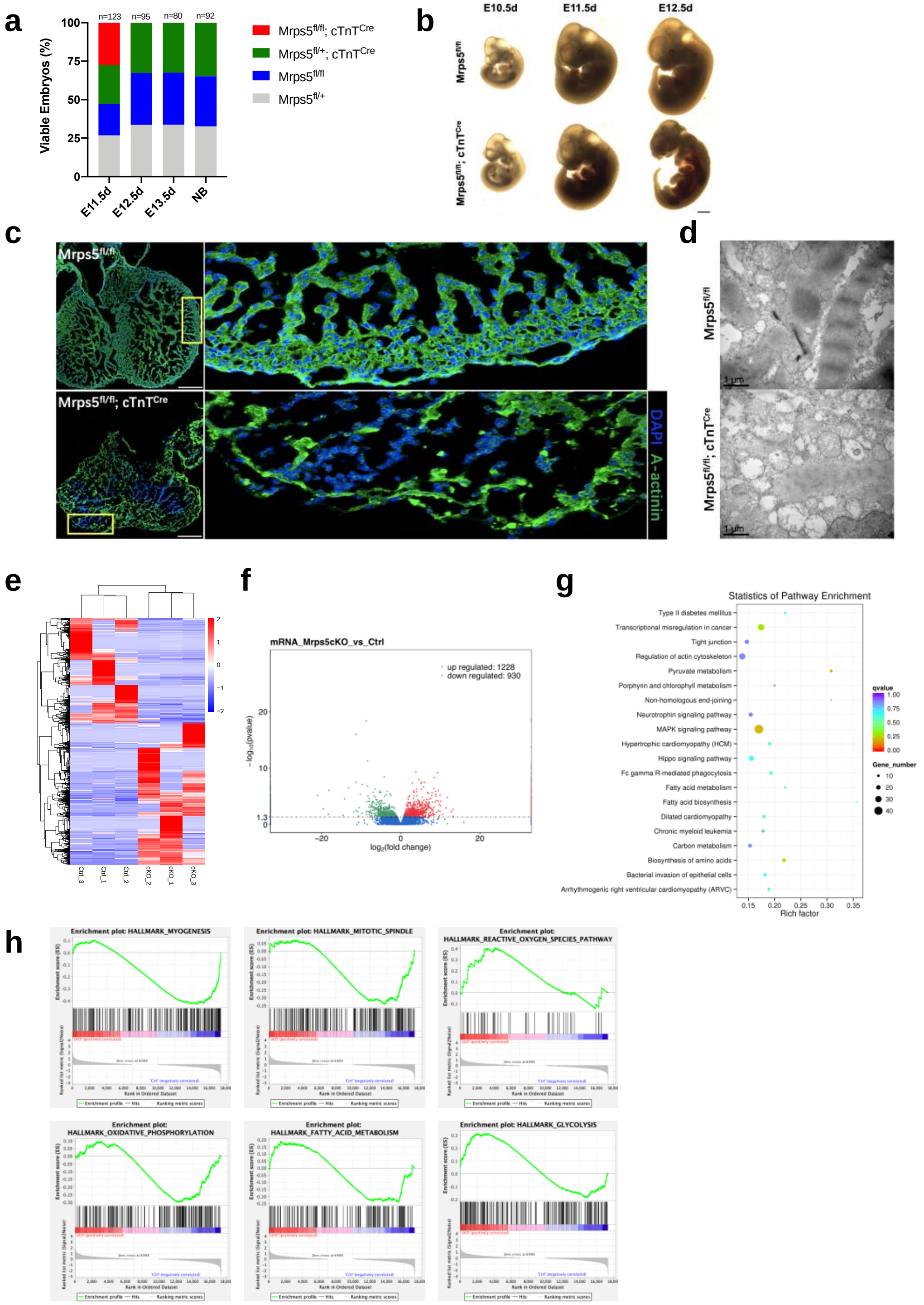

Supplementary Figure 2. Cardiomyocyte-specific *Mrps5* deletion during embryogenesis results in abnormal heart development. a. Distribution of genotypes at indicated gestational ages. NB, newborn. **b.** Morphology of *Mrps5*$^{fl/fl}$ and *Mrps5*$^{fl/fl}$;cTnT^{Cre} mice at E10.5, E11.5 and E12.5. Scale bar = 200 μm. **c.** Representative E12 heart sections stained for α-actinin and DAPI. Scale bar = 100 μm. **d.** Representative images of mitochondria in cardiomyocyte of *Mrps5*$^{fl/fl}$ and *Mrps5*$^{fl/fl}$;cTnT^{Cre} hearts at E12. Scale bar = 1 μm. **e.** Heatmap of dysregulated transcripts in *Mrps5*$^{fl/fl}$ and *Mrps5*$^{fl/fl}$;cTnT^{Cre} hearts at E12. **f.** Volcano plot representing dysregulated transcripts in *Mrps5*$^{fl/fl}$;cTnT^{Cre} hearts compared with *Mrps5*$^{fl/fl}$ and at E12. **g.** KEGG enrichment analysis showing dysregulated signaling pathways. **h.** Representative dysregulated signaling pathways from Gene set enrichment analysis (GSEA). P values were determined by 2-tailed unpaired Students' *t*-test in (F).

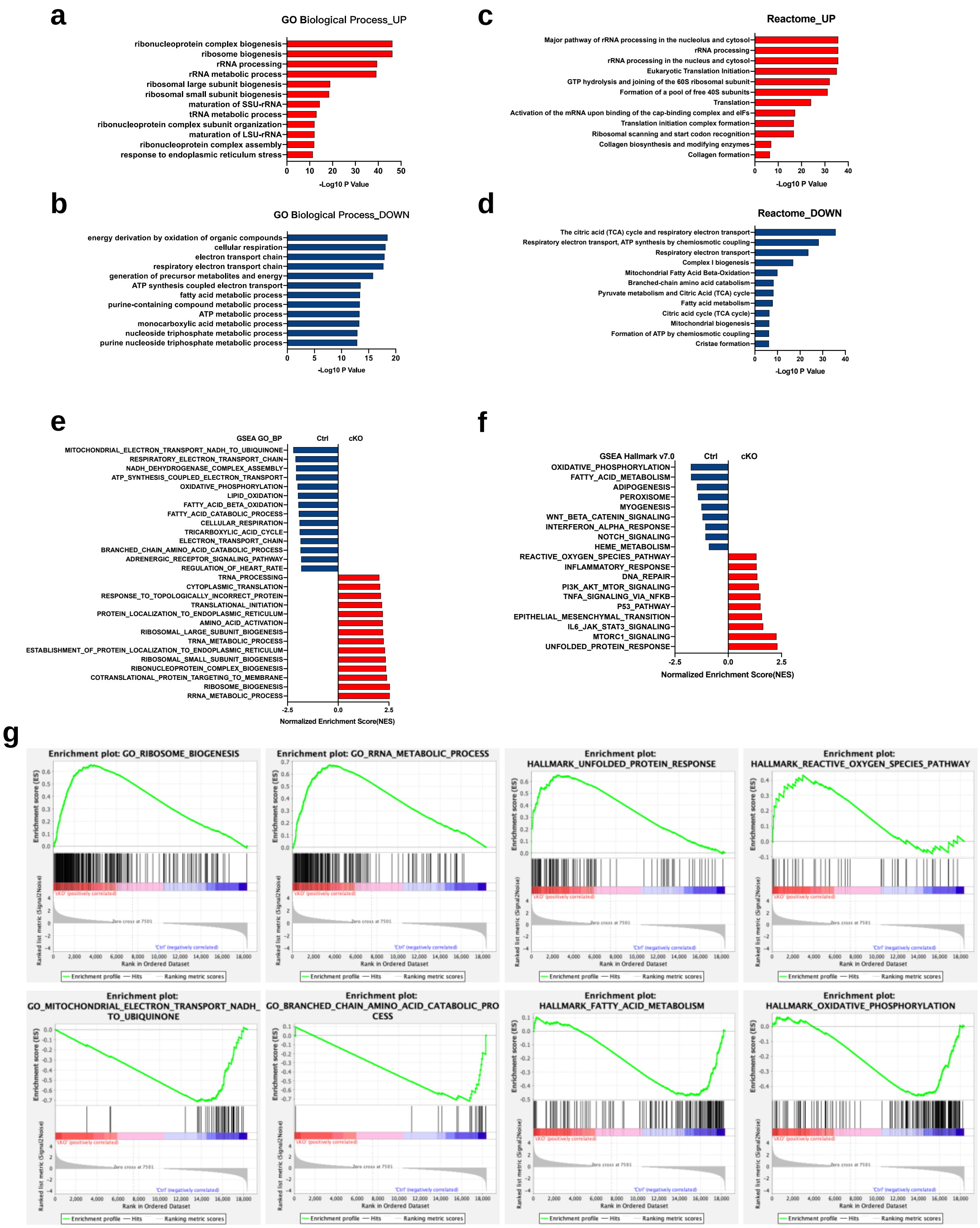

Supplementary Figure 3. Loss of *Mrps5* alters translational and metabolic programs in the heart. a. Gene Ontology (GO) annotation of the biological processes associated with upregulated genes in *Mrps5*^{cKO} hearts compared with *Mrps5*$^{fl/fl}$. **b.** Gene Ontology (GO) annotation of the biological processes associated with downregulated genes in *Mrps5*^{cKO} hearts compared with *Mrps5*$^{fl/fl}$. **c.** Reactome database analysis of the upregulated genes in *Mrps5*^{cKO} hearts compared with *Mrps5*$^{fl/fl}$. **d.** Reactome database analysis of the downregulated genes in *Mrps5*^{cKO} hearts compared with *Mrps5*$^{fl/fl}$. **e.** Gene set enrichment analysis (GSEA) with GO database showing dysregulated biological processes. **f.** Gene set enrichment analysis (GSEA) with hallmark database showing dysregulated pathways. **g.** Representative dysregulated signaling pathways from GSEA. P values were determined by 2-tailed unpaired Students' *t*-test in **a-d**.

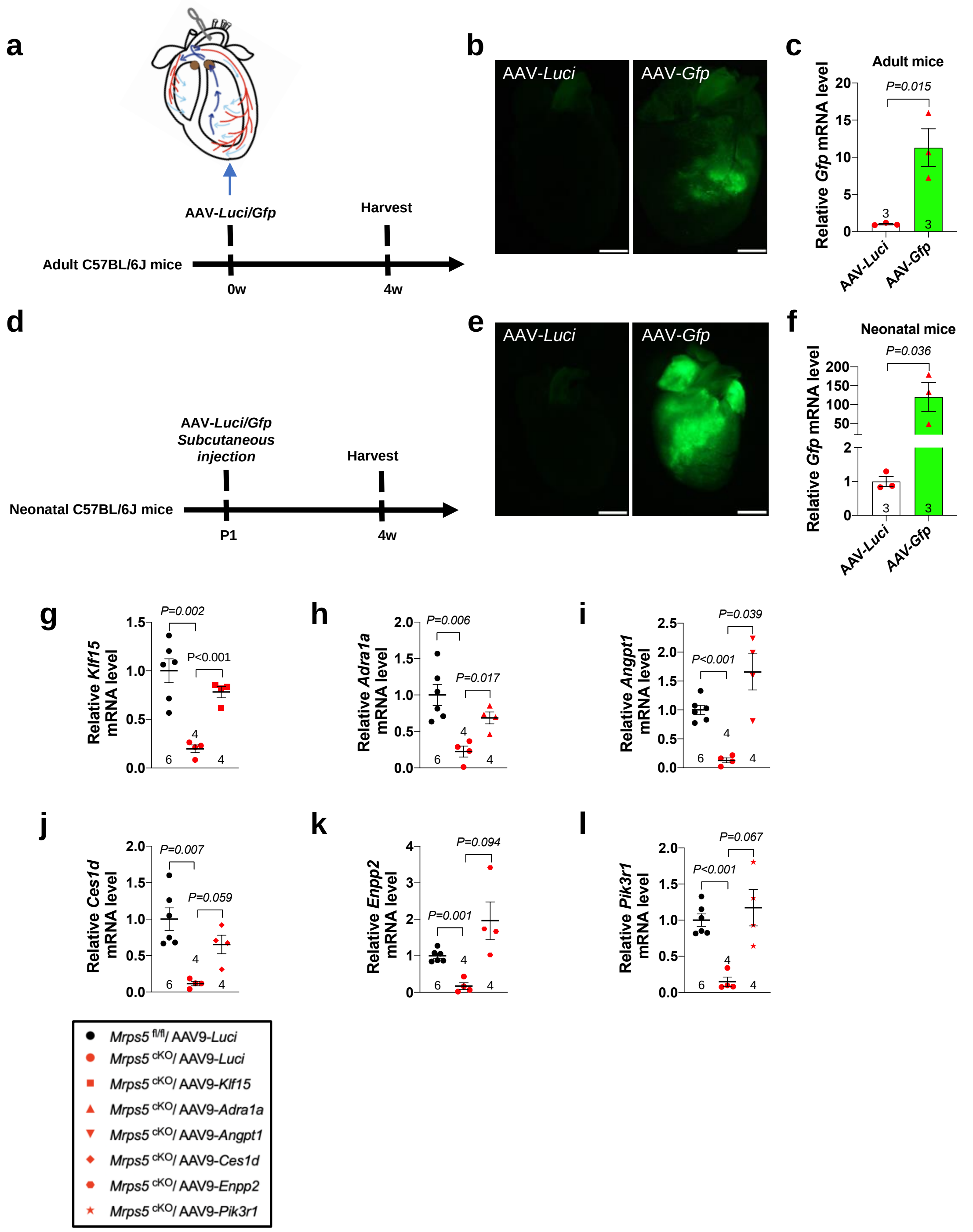

Supplementary Figure 4. AAV9 mediated target gene expression delivery system. a. Schematic depiction of AAV9-*Luci*/*Gfp* delivery method into adult mouse heart tissue. **b.** Representative images of adult mouse hearts infected with AAV-*Luci* and AAV-*Gfp* at 4 weeks post AAV9 injection. Scale bar = 500 μm. **c.** *Gfp* transcript expression in adult mouse heart infected with AAV-*Luci* and AAV-*Gfp* at 4 weeks post AAV9 injection. **d.** Schematic depiction of AAV9-*Luci*/*Gfp* delivery method into neonatal mouse heart. **e.** Representative images of neonatal mouse hearts infected with AAV-*Luci* and AAV-*Gfp* at 4 weeks post AAV9 injection. Scale bar = 500 μm. **f.** *Gfp* transcript expression in neonatal mouse heart infected with AAV-*Luci* and AAV-*Gfp* at 4 weeks post AAV9 injection. **g-l.** Gene expression level of *Klf15*, *Adra1a*, *Angpt1*, *Ces1d*, *Enpp2* and *Pik3r1* from each group as indicated at 14 weeks after tamoxifen injection and following AAV9 induced target gene expression for 7 weeks. N numbers are indicated in each panel. All data are presented as mean ± SEM. P values were determined by 2-tailed unpaired Students' *t*-test in **c, f.** P values were determined by one-way ANOVA with Brown-Forsythe and Welch multiple comparisons test in **g-l.**

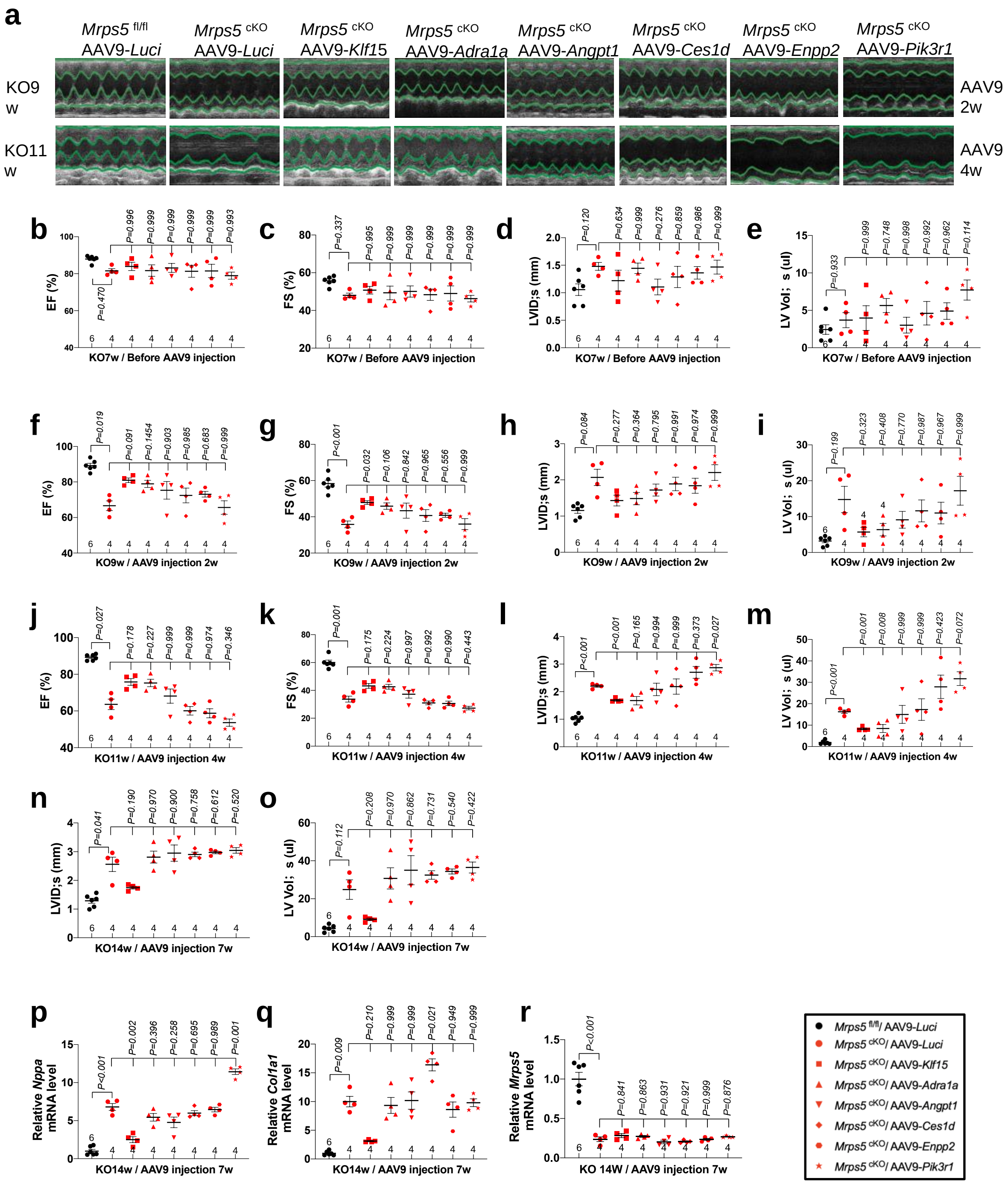

Supplementary Figure 5. AAV9 mediated target gene re-expression protects *Mrps5*^{cKO} mouse heart function. a. Representative images of M-mode echocardiography of mice at 9 w (AAV treated for 2w) and 11w (AAV treated for 4w) after tamoxifen injection for each group, as indicated. **b-e.** Quantification of EF, FS, LVID;s, LV vol;s at 7 weeks after tamoxifen injection prior to AAV9 injection, for each group, as indicated in Figure. **f-i.** Quantification of EF, FS, LVID;s, LV vol;s at 9 weeks after tamoxifen injection with AAV9 induced target gene expression for a duration of 2 weeks. **j-m.** Quantification of EF, FS, LVID;s, LV vol;s at 11 weeks after tamoxifen injection with AAV9 induced target gene expression for a duration of 4 weeks. **n-o.** Quantification of LVID;s and LV vol;s at 14 weeks after tamoxifen injection with AAV9 induced target gene expression for a duration of 7 weeks. **p-r.** Gene expression level of *Nppa*, *Col1a1* and *Mrps5* from each group as indicated at 14 weeks after tamoxifen injection with AAV9 induced target gene expression for a duration of 7 weeks. N numbers are indicated in each panel. All data are presented as mean ± SEM. P values were determined by one-way ANOVA with Brown-Forsythe and Welch multiple comparisons test.

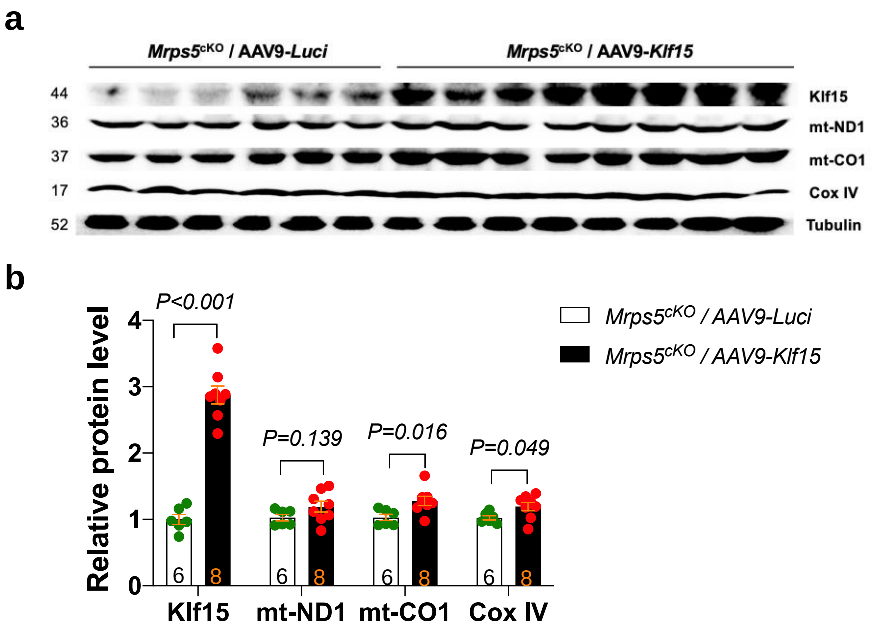

Supplementary Figure 6. Re-expression of Klf15 in the heart restores mitochondrial ETC proteins in *Mrps5*^{cKO} mutant mice. a. Western blot detecting KLF15, mt-ND1, mt-CO1, Cox IV and Tubulin proteins from *Mrps5*^{cKO} hearts infected with control AAV9-*Luci* (n=6) or AAV9-*Klf15* virus (n=8). **b.** Quantification of Western blot results. P values were determined by 2-tailed unpaired Students' *t*-test.

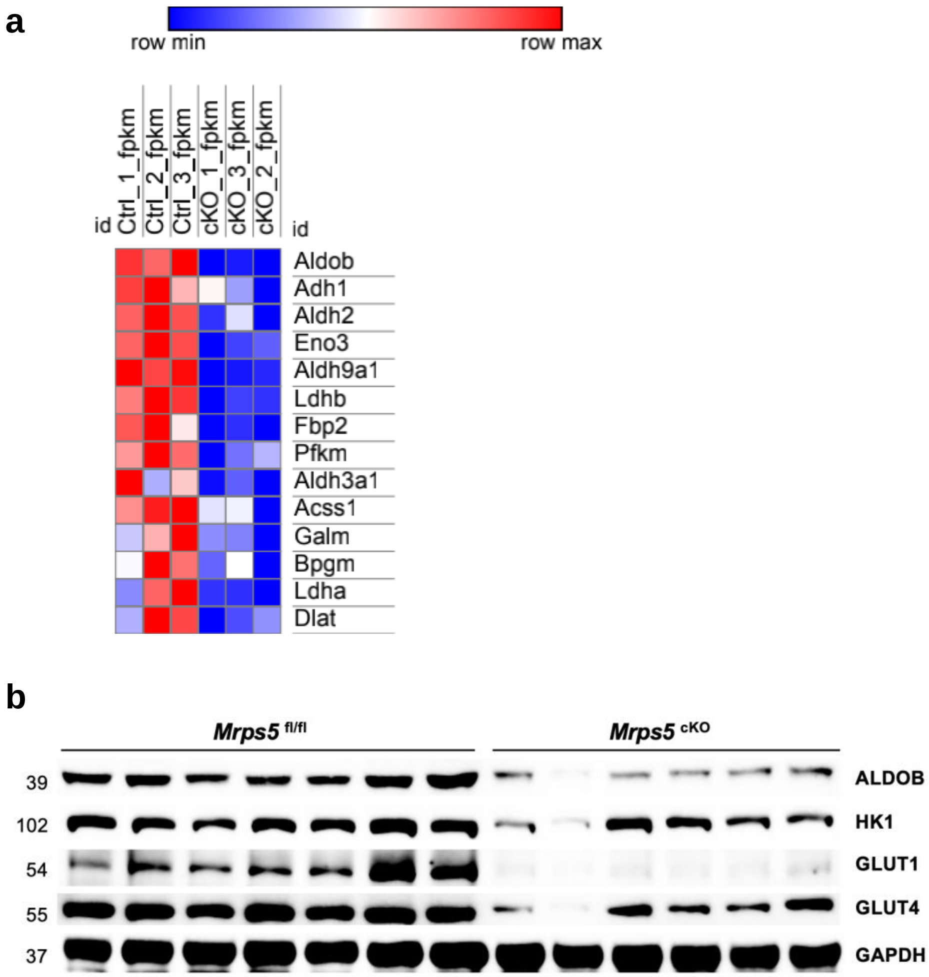

Supplementary Figure 7. Glycolysis associated genes and proteins decreased in *Mrps5*^{cKO} hearts. a. Heatmap of the decreased glycolysis associated genes in *Mrps5*^{cKO} hearts. **b.** Representative immunoblot images of ALDOB, HK1, GLUT1, GLUT4 and GAPDH in control and *Mrps5*^{cKO} hearts 12 weeks after *Mrps5* gene deletion.

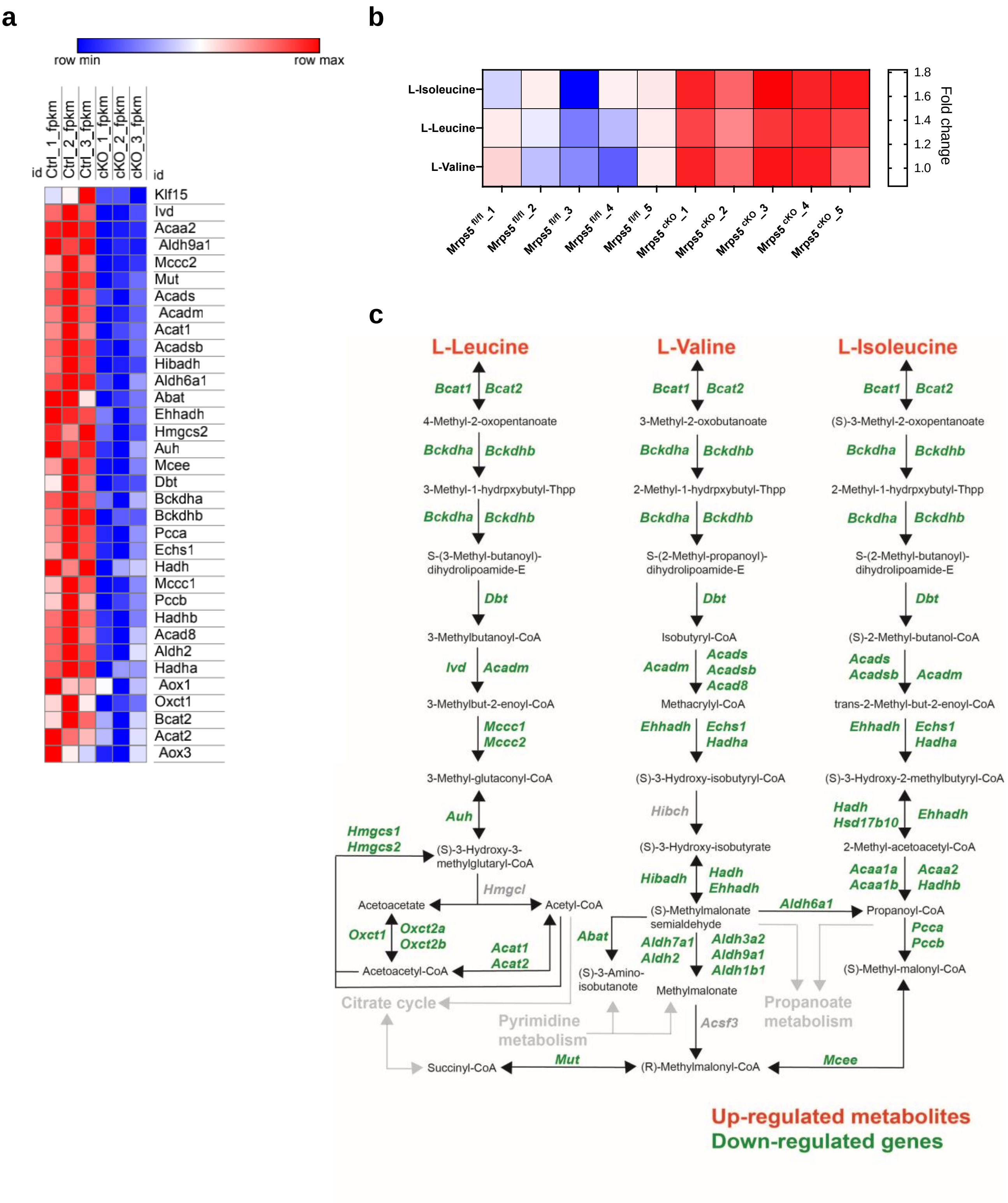

Supplementary Figure 8. BCAAs catabolism is decreased in *Mrps5*^{cKO} hearts. a. Heatmap of the decreased BCAAs catabolism associated genes in *Mrps5*^{cKO} hearts. **b.** Increased BCAAs and decreased BCAAs catabolism associated genes in *Mrps5*^{cKO} hearts. **c.** Overview of BCAA catabolism pathways illustrating elevated BCAAs and downregulated catabolism genes detected in *Mrps5*^{cKO} hearts.

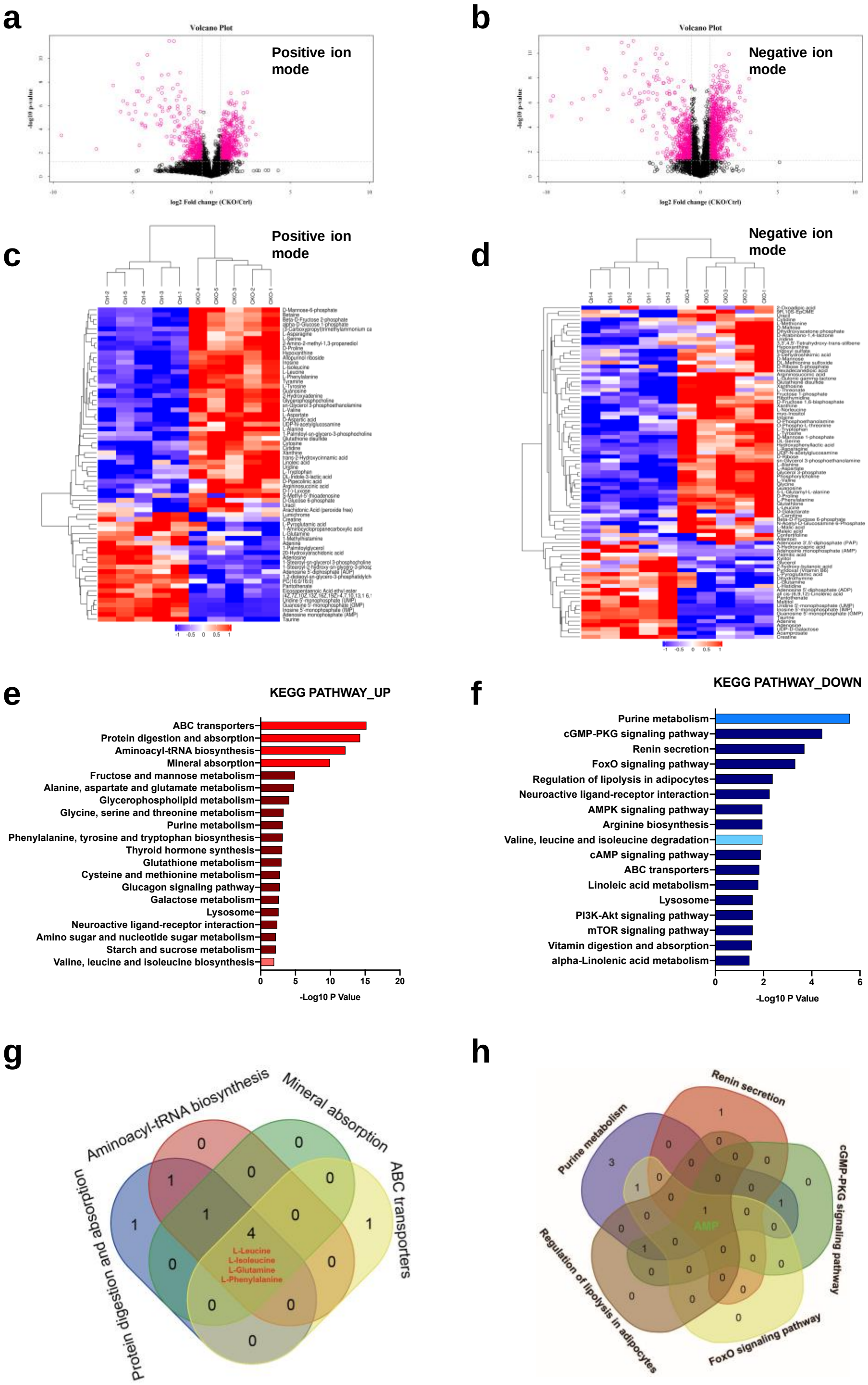

Supplementary Figure 9. Unbiased metabolomics analysis reveals the metabolite changes in *Mrps5*^{cKO} hearts. a. Volcano plot of the dysregulated metabolites in *Mrps5*^{cKO} hearts (positive ion). **b.** Volcano plot of the dysregulated metabolites in *Mrps5*^{cKO} hearts (negative ion). **c.** Heatmap of the dysregulated metabolites in *Mrps5*^{cKO} hearts (positive ion). **d.** Heatmap of the dysregulated metabolites in *Mrps5*^{cKO} hearts (negative ion). **e.** KEGG functional enrichment analysis of the upregulated metabolites in *Mrps5*^{cKO} hearts. **f.** KEGG functional enrichment analysis of the downregulated metabolites in *Mrps5*^{cKO} hearts. **g.** Venn diagram illustrating the top four enriched pathways of upregulated metabolites in *Mrps5*^{cKO} hearts. **h.** Venn diagram illustrating the top five enriched pathways of downregulated metabolites in *Mrps5*^{cKO} hearts. P values were determined by 2-tailed unpaired Students' *t*-test in **a, b, e, f**.

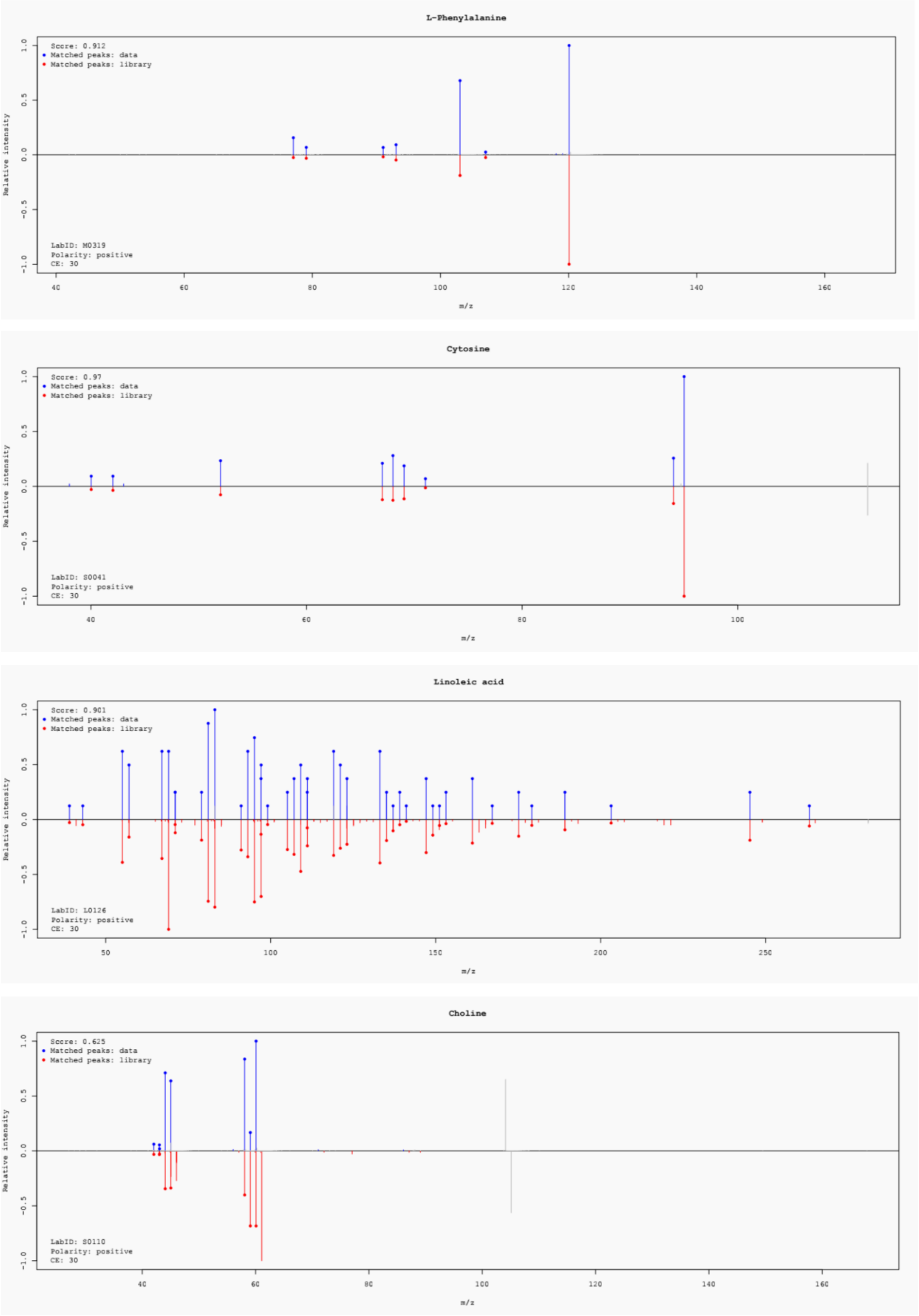

Supplementary Figure 10. Upregulated metabolites in *Mrps5*^{cKO} hearts. Raw data from analysis of *Mrps5*^{cKO} hearts indicating upregulated metabolites.

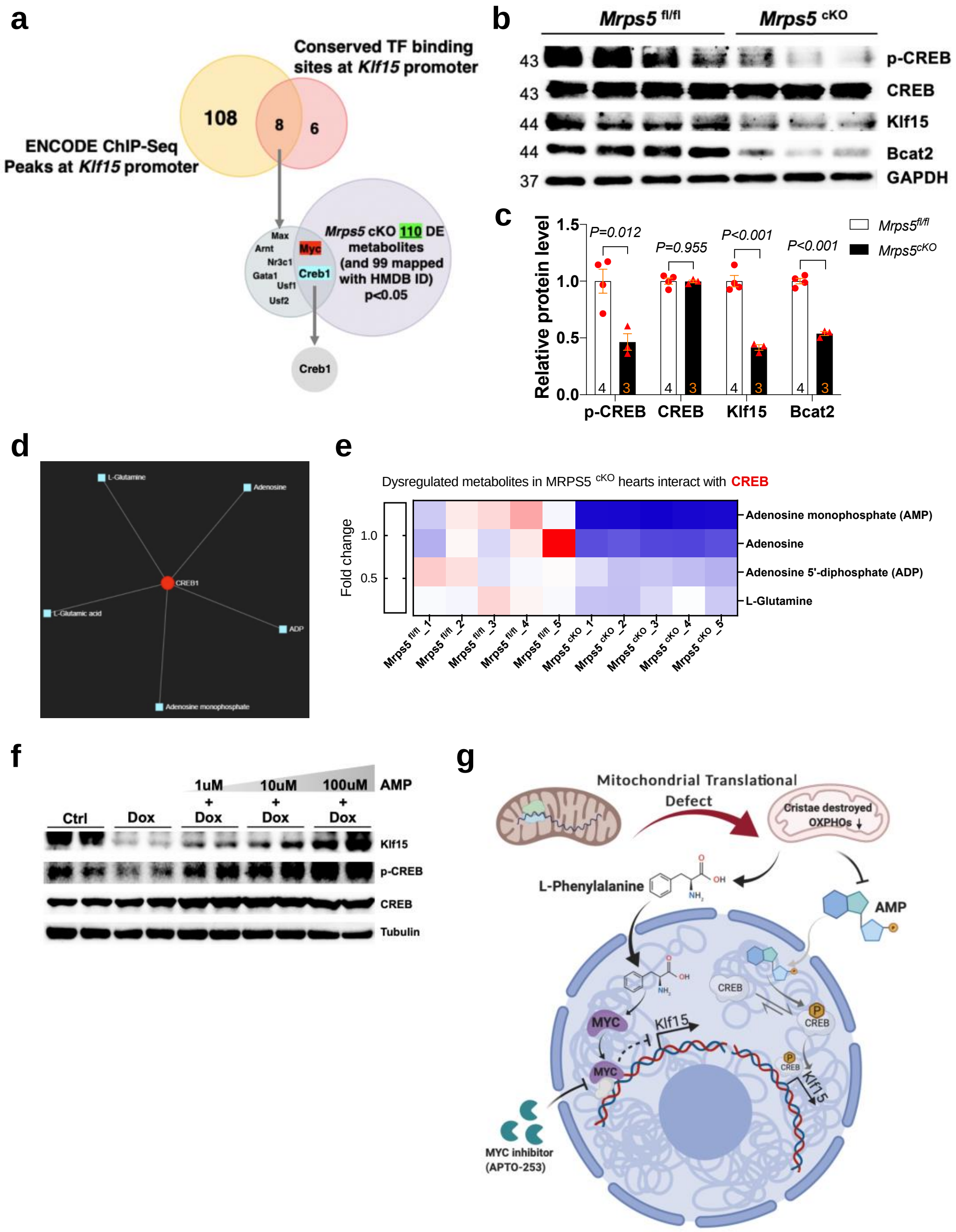

Supplementary Figure 11. AMP/p-CREB signaling axis functions as another upstream mediator of Klf15 expression in *Mrps5*^{cKO} hearts. a. Rational used to identify potential upstream signals regulating Klf15 expression in *Mrps5*^{cKO} hearts. Analysis of ENCODE ChIP-Sequence peaks and conserved transcription factor binding sites over the *Klf15* promoter were determined. This information was correlated with the untargeted metabolomics data from *Mrps5*^{cKO} hearts to determine candidates for upstream metabolite interaction. **b.** Representative immunoblot with whole heart lysates from *Mrps5*$^{fl/fl}$ (n=4) and *Mrps5*^{cKO} mice (n=3). Examined proteins as indicated in Figure. **c.** Quantification of protein levels determined from immunoblot in **b**. **d.** Interaction networks of *Creb1* and DE metabolites after *Mrps5* deletion. **e.** Heatmap of DE metabolites shown to interact with *Creb1* after *Mrps5* deletion. *n*=5 per condition. **f.** Representative immunoblot of whole cell lysates from H9C2 cells after doxycycline or doxycycline + AMP treatment for 72h. **g.** Schematic depiction of regulatory network for Klf15 expression by L-phenylalanine/MYC and AMP/p-CREB signaling axes in context of mitochondrial translational defect. Created with BioRender.com. All data are presented as mean ± SEM. P values were determined by 2-tailed unpaired Students' *t*-test.

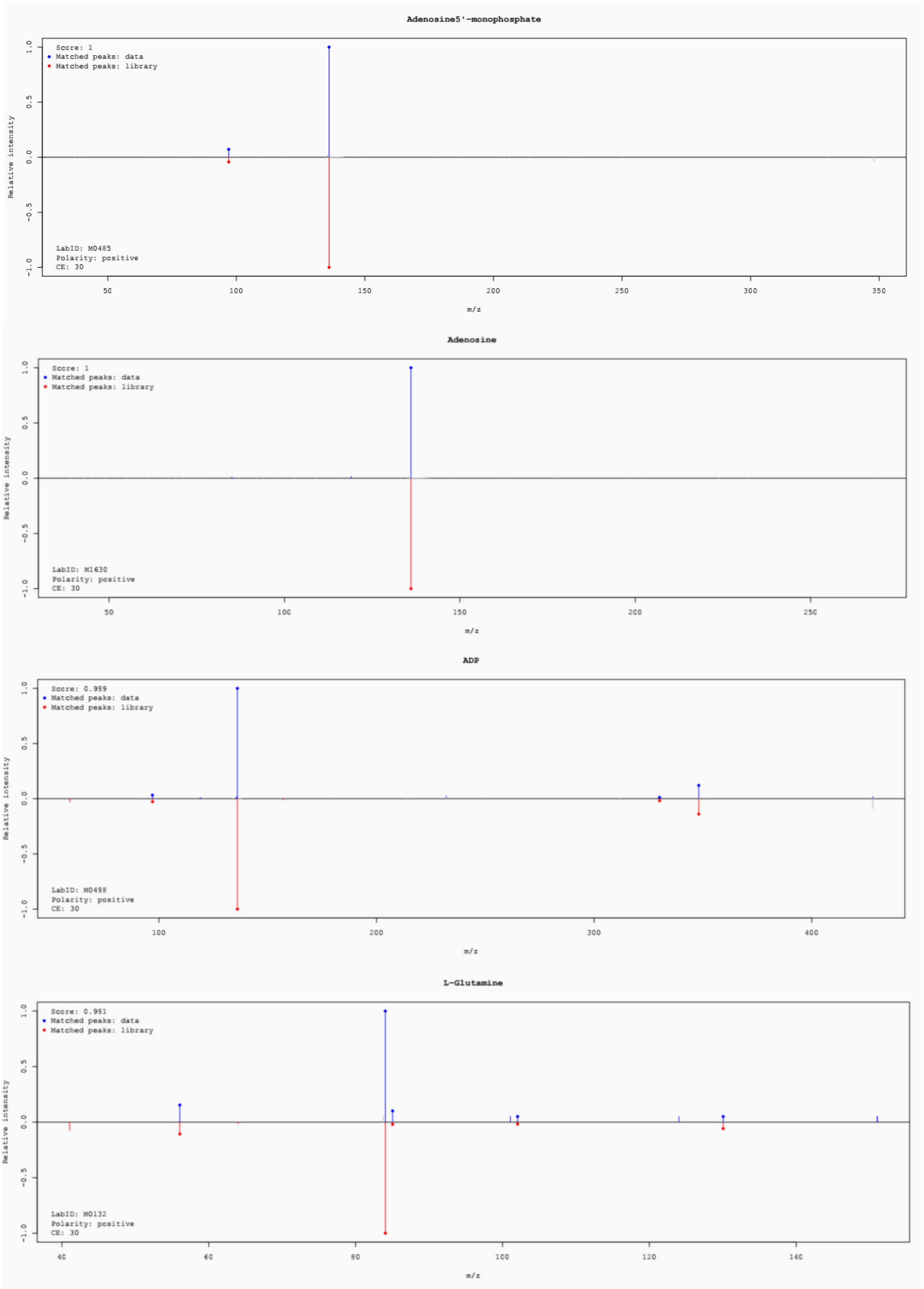

Supplementary Figure 12. Downregulated metabolites in *Mrps5*^{cKO} hearts. Raw data from analysis of *Mrps5*^{cKO} hearts indicating downregulated metabolites.

Sample	Gender	Age	Diagnosis
**Ctrl_1**	Male	52	Donor (normal heart)
**Ctrl_2**	Male	15	Donor (normal heart)
**Ctrl_3**	Male	67	Donor (normal heart)
**DCM_1**	Male	48	Right ventricular dilated cardiomyopathy (with hypertension, diabetes, liver cirrhosis)
**DCM_2**	Male	62	Dilated cardiomyopathy
**DCM_3**	Male	49	Dilated cardiomyopathy （with cardiac valve diseases, arrhythmia, hypertension）
**DCM_4**	Male	68	Dilated cardiomyopathy （with cardiac valve diseases, pulmonary hypertension, atrial fibrillation）
**DCM_5**	Male	41	Dilated cardiomyopathy (heart failure)
**DCM_6**	Female	34	Dilated cardiomyopathy

Supplementary Table 1. Clinical characterization of patient samples used in this study. Sources of human tissue used for analysis of the expression of human *Mrps5*. Levels of *Mrps5* were decreased in the heart tissue samples from patients with dilated cardiomyopathy.

Gene set	TF candidates regulate Klf15
**ENCODE ChiP-Seq Peaks at *Klf15* promoter**	Myc Max Mxi1 Mga Ep300 Gata2 Bach1 Cbfa2t3 Mitf Nfyb Rxra Tbx3 Ctcf Jund Ncor1 Rbfox2 Xrcc5 Zmym3 Ago2 Atf3 Atf4 Esr1 Phf6 Smarca4 Taf15 Meis2 Arnt Nr3c1 Gata1 Creb1 Usf1 Usf2 E2f6 Tal1 Tcf12 Bhlhe40 Znf31b Zbtb7a Cebpb Kdm4a Suz12 Pknox1 Phf21a Nanog Znf143 Ctbp1 Foxm1 Neuprod1 Ncoa1 Prdm10 Foxa1 Smad1 Rbm39 Mynn Elf1 Taf1 Foxk2 Rcor1 Foxp1 Esrra Arid3a Taf9b Jun Cbfa2t2 Sox6 Gmeb1 Ubtf Irf1 Egr1 Crem Nr2f2 U2af1 Rad21 Hnrnpl Hnf4a Hnf4g Foxa2 E4f1 Sin3a Rest Kdm1a Gabpa Mbd2 Rbm22 Zbtb33 Rnf2 Hdac1 Hnrnpll Sp1 Atf7 Rad51 L3mbtl2 Zfx Nrf1 Ezh2 Mnt Hdac2 Yy1 Ago1 Polr2g Ep400 Znf316 Ets1 Ash2l Znf24 Polr2a Ski Lef1 Tbp Znf579 Znf217 Mafk Gata3 Trim28 Sap30 Rfx5 Thap1
**Conserved TF binding sites at *Klf15* promoter**	Myc Max Ahr Arnt Nr3c1 Gata1 Creb1 Usf1 Usf2 Gfi1 Hox13 Srebp1 Mzf1 Mycn
**Overlapped with *Mrps5* cKO DEG (adj p<0.05)**	Myc Max Ahr Mxi1 Mga Ep300 Gata2 Bach1 Cbfa2t3 Mitf Nfyb Rxra Tbx3 Ctcf Jund Ncor1 Rbfox2 Xrcc5 Zmym3 Ago2 Atf3 Atf4 Esr1 Phf6 Smarca4 Taf15 Meis2

Supplementary Table 2. Candidate transcription factors for regulation of Klf15. Top Panel, Gene list determined from ENCODE ChIP-Seq analysis over the Klf15 promoter. Middle Panel, Conserved TF binding sites on the Klf15 promoter based on sequence comparison between species. Bottom Panel, List of overlapping genes upon comparison of DEG genes identified in *Mrps5*^{cKO} hearts (Figure 2) and ENCODE ChIP-Seq analysis from top panel. The genes indicated in red are conserved in all three groups/panels. The genes indicated in purple are conserved in both the ENCODE ChIP-Seq data and list of conserved TF binding sites on the *Klf15* promoter. The genes indicated in green were present in both the list of ENCODE ChIP-Seq peaks and in the list of DEG genes. The gene indicated in yellow was conserved in the list of DEG genes and list of conserved TF binding sites. The eight transcriptional regulators of *Klf15* referred to in the text consist of the genes in red and purple.

Pathway	Total	Expected	Hits	Raw p	-LOG10(p)	Holm adjust	FDR	Impact
Aminoacyl-tRNA biosynthesis	118	5.437	35	1.89E-19	18.725	6.17E-17	6.17E-17	0.46392
Protein digestion and absorption	141	6.4967	29	7.29E-12	11.137	2.38E-09	1.19E-09	0
Central carbon metabolism in cancer	104	4.7919	24	3.75E-11	10.426	1.22E-08	4.09E-09	0.17857
ABC transporters	186	8.5701	32	8.63E-11	10.064	2.80E-08	7.05E-09	0
Mineral absorption	81	3.7322	19	3.12E-09	8.5061	1.01E-06	2.04E-07	0.023529
Glycine, serine and threonine metabolism	90	4.1468	18	1.14E-07	6.9428	3.67E-05	6.22E-06	1.4318
Glutathione metabolism	103	4.7458	18	9.45E-07	6.0245	0.00030341	4.42E-05	0.86957
Purine metabolism	231	10.644	27	8.00E-06	5.0971	0.0025588	0.000327	1.8382
Hepatocellular carcinoma	186	8.5701	22	4.56E-05	4.3407	0.014558	0.001658	0.24576
ECM-receptor interaction	89	4.1008	14	5.18E-05	4.2859	0.016462	0.001693	0.49306
HIF-1 signaling pathway	127	5.8517	17	7.10E-05	4.1487	0.022509	0.0021	0.64634
Platinum drug resistance	81	3.7322	13	7.71E-05	4.1132	0.02435	0.0021	0.3
p53 signaling pathway	71	3.2714	12	8.63E-05	4.0639	0.027192	0.002171	0.34375

Supplementary Table 3. MetaboAnalyst integrative transcriptome and metabolome analysis. Analyses were performed to determine the dysregulated transcriptome and metabolome pathways in *Mrsp5*^{cKO} hearts. Pathways are listed with the most significantly altered at the top (based on P value).
