# Supplementary material for: A defect in mitochondrial protein translation influences mitonuclear communication in the heart
Source: Nat Commun. 2023 Mar 22;14:1595. doi: 10.1038/s41467-023-37291-5 (PMC10033703; doi:10.1038/s41467-023-37291-5)
Supplement: Supplementary file 2 — Reporting Summary [file 41467_2023_37291_MOESM2_ESM.pdf]

nature portfolio

Corresponding author(s): Da-Zhi Wang, Jinghai Chen, and Jian'an Wang

Last updated by author(s): Feb 16, 2023

Reporting Summary

Nature Portfolio wishes to improve the reproducibility of the work that we publish. This form provides structure for consistency and transparency in reporting. For further information on Nature Portfolio policies, see our Editorial Policies and the Editorial Policy Checklist.

Statistics

For all statistical analyses, confirm that the following items are present in the figure legend, table legend, main text, or Methods section.

n/a	Confirmed	
☐	☒	The exact sample size (*n*) for each experimental group/condition, given as a discrete number and unit of measurement
☐	☒	A statement on whether measurements were taken from distinct samples or whether the same sample was measured repeatedly
☐	☒	The statistical test(s) used AND whether they are one- or two-sided<br>*Only common tests should be described solely by name; describe more complex techniques in the Methods section.*
☐	☒	A description of all covariates tested
☐	☒	A description of any assumptions or corrections, such as tests of normality and adjustment for multiple comparisons
☐	☒	A full description of the statistical parameters including central tendency (e.g. means) or other basic estimates (e.g. regression coefficient) AND variation (e.g. standard deviation) or associated estimates of uncertainty (e.g. confidence intervals)
☐	☒	For null hypothesis testing, the test statistic (e.g. *F*, *t*, *r*) with confidence intervals, effect sizes, degrees of freedom and *P* value noted<br>*Give P values as exact values whenever suitable.*
☐	☒	For Bayesian analysis, information on the choice of priors and Markov chain Monte Carlo settings
☐	☒	For hierarchical and complex designs, identification of the appropriate level for tests and full reporting of outcomes
☐	☒	Estimates of effect sizes (e.g. Cohen's *d*, Pearson's *r*), indicating how they were calculated

Our web collection on statistics for biologists contains articles on many of the points above.

Software and code

Policy information about availability of computer code

Data collection	Visual Sonics Vevo 2100 Imaging System software
Data analysis	Image J version 1.52a, Image Lab version 6.0.1, GraphPad Prism version 9.0.1

For manuscripts utilizing custom algorithms or software that are central to the research but not yet described in published literature, software must be made available to editors and reviewers. We strongly encourage code deposition in a community repository (e.g. GitHub). See the Nature Portfolio guidelines for submitting code & software for further information.

Data

Policy information about availability of data

All manuscripts must include a data availability statement. This statement should provide the following information, where applicable:
- Accession codes, unique identifiers, or web links for publicly available datasets
- A description of any restrictions on data availability
- For clinical datasets or third party data, please ensure that the statement adheres to our policy

RNA seq data has been deposited in GEO under accession codes: GSE200940, GSE200999, and GSE201022. All data and reagents described in this manuscript will be administered in accordance with both the University of South Florida (USF) and NIH policies, including the NIH Data Sharing Policy and Implementation Guidance of March 5, 2003.

Human research participants

Policy information about studies involving human research participants and Sex and Gender in Research.

Reporting on sex and gender	Not collected.
Population characteristics	Human cardiac tissue biopsies were obtained from patients of the Second Affiliated Hospital Zhejiang University School of Medicine. Samples were obtained from 6 patients with DCM (average age 50 years old) and 3 patients without DCM (average age 45 years old).
Recruitment	The patients with DCM were recruited while in hospital undergoing heart transplantation surgery. The patients without DCM were donors with healthy hearts. The sex and age were not considered in the analysis.
Ethics oversight	All experimental procedures involving humans were approved by the Ethics Committee of Second Affiliated Hospital of Zhejiang University. Patients provided written informed consent.

Note that full information on the approval of the study protocol must also be provided in the manuscript.

Field-specific reporting

Please select the one below that is the best fit for your research. If you are not sure, read the appropriate sections before making your selection.

☒ Life sciences ☐ Behavioural & social sciences ☐ Ecological, evolutionary & environmental sciences

For a reference copy of the document with all sections, see nature.com/documents/nr-reporting-summary-flat.pdf

Life sciences study design

All studies must disclose on these points even when the disclosure is negative.

Sample size	Sample sizes were based on similar published studies. For in vivo studies, at least 3 mice were used for each condition. The number of the independent replicates is indicated in each figure. At least 3 experimental replicates were used unless otherwise noted.
Data exclusions	None
Replication	All in vivo and in vitro experiments were independently performed at least 3 times. All attempts at replication were successful.
Randomization	For experiments employing wild-type mice, animals were randomized based on body weight. For experiments using genetically modified mice, animals were grouped through their genotype. For in vitro study, cells were grown under the same conditions and randomly allocated into different groups without bias.
Blinding	The investigators were blinded to group allocation during data collection and analysis. We collected and analyzed the samples under these same conditions.

Reporting for specific materials, systems and methods

We require information from authors about some types of materials, experimental systems and methods used in many studies. Here, indicate whether each material, system or method listed is relevant to your study. If you are not sure if a list item applies to your research, read the appropriate section before selecting a response.

Materials & experimental systems

n/a	Involved in the study	
☐	☒	Antibodies
☐	☒	Eukaryotic cell lines
☒	☐	Palaeontology and archaeology
☐	☒	Animals and other organisms
☒	☐	Clinical data
☒	☐	Dual use research of concern

Methods

n/a	Involved in the study	
☒	☐	ChIP-seq
☒	☐	Flow cytometry
☒	☐	MRI-based neuroimaging

Antibodies

Antibodies used	MRPS5 protein was detected with rabbit antibody to MRPS5 (Gene Tex, GTX103930; 1:1000 dilution);<br>mt-ATP6 protein was detected with mouse antibody to mt-ATP6 (Abcam, ab219825; 1:1000 dilution);<br>mt-CO1 protein was detected with rabbit antibody to mt-CO1 (Abcam, ab203912; 1:1000 dilution);<br>mt-ND1 protein was detected with rabbit antibody to mt-ND1 (Abcam, ab181848; 1:1000 dilution);<br>VDAC protein was detected with rabbit antibody to VDAC (CST, 4661S; 1:1000 dilution);<br>p-CREB protein was detected with rabbit antibody to p-CREB (CST, 9198S; 1:1000 dilution);<br>CREB protein was detected with rabbit antibody to CREB (CST, 9197S; 1:1000 dilution);<br>KLF15 protein was detected with rabbit antibody to KLF15 (Abcam, ab2647; 1:1000 dilution);<br>BCAT2 protein was detected with rabbit antibody to BCAT2 (Abcam, ab95976; 1:1000 dilution);<br>MYC protein was detected with rabbit antibody to MYC (CST, 9402S; 1:1000 dilution);<br>ALDOB protein was detected with rabbit antibody to ALDOB (HUABIO, ER62642; 1:1000 dilution);<br>β-Actin protein was detected with mouse antibody to β-Actin (HUABIO, EM2001-07; 1:5000 dilution);<br>HK1 protein was detected with rabbit antibody to HK1 (HUABIO, ST47-05; 1:1000 dilution);<br>GLUT1 protein was detected with rabbit antibody to GLUT1 (HUABIO, ET1601-10; 1:1000 dilution);<br>GLUT4 protein was detected with rabbit antibody to GLUT4 (HUABIO, R1402-3; 1:1000 dilution);<br>GAPDH protein was detected with rabbit antibody to GAPDH (HUABIO, R1210-1; 1:5000 dilution);
Validation	Protein bands were visualized with the Bio-Rad ChemiDoc imaging system. Validated by manufacturer and in cited publications.<br>MRPS5 (Gene Tex, GTX103930); https://www.ncbi.nlm.nih.gov/pubmed/30566640<br>mt-ATP6 (Abcam, ab219825); https://www.ncbi.nlm.nih.gov/pubmed/32042910<br>mt-CO1 (Abcam, ab203912); https://www.ncbi.nlm.nih.gov/pubmed/33735837<br>mt-ND1 (Abcam, ab181848); https://www.ncbi.nlm.nih.gov/pubmed/33279600<br>VDAC (CST, 4661S); https://www.ncbi.nlm.nih.gov/pubmed/29578301<br>p-CREB (CST, 9198S); https://www.ncbi.nlm.nih.gov/pubmed/33203971<br>CREB (CST, 9197S); https://www.ncbi.nlm.nih.gov/pubmed/35835749<br>KLF15 (Abcam, ab2647); https://www.ncbi.nlm.nih.gov/pubmed/30830866<br>BCAT2 (Abcam, ab95976); https://www.ncbi.nlm.nih.gov/pubmed/25050624<br>MYC (CST, 9402S); https://www.ncbi.nlm.nih.gov/pubmed/33298911<br>ALDOB (HUABIO, ER62642); http://www.huabio.cn/search?sort=asc&keyword=ER62642<br>β-Actin (HUABIO, EM2001-07); http://www.huabio.cn/search?sort=asc&keyword=EM2001-07<br>HK1 (HUABIO, ST47-05); https://www.huabio.com/products/hexokinase-1-antibody-clone-st47-05-recombinant-monoclonal-et1609-28<br>GLUT1 (HUABIO, ET1601-10); https://pubmed.ncbi.nlm.nih.gov/34922943<br>GLUT4 (HUABIO, R1402-3); https://pubmed.ncbi.nlm.nih.gov/36297098<br>GAPDH (HUABIO, R1210-1); https://pubmed.ncbi.nlm.nih.gov/35115492

Eukaryotic cell lines

Policy information about cell lines and Sex and Gender in Research

Cell line source(s)	293T cell line, derived from human embryonic kidney
Authentication	Cell lines were not independently authenticated.
Mycoplasma contamination	Cell lines used tested negative for Mycoplasma contamination.
Commonly misidentified lines (See ICLAC register)	No commonly misidentified cell lines were used in this study.

Animals and other research organisms

Policy information about studies involving animals; ARRIVE guidelines recommended for reporting animal research, and Sex and Gender in Research

Laboratory animals	Animals used were all male, aged eight weeks and postnatal day 1. The strains included aMHC-MCM, cTnT-Cre, Mrps5 (flox/flox) and C57BL/6J wild type mice. Mice were housed on a 12-h light / dark cycle and given free access to food and water at a temperature of 18-23 degrees C, humidity at 40-60%.
Wild animals	The study did not involve wild animals.
Reporting on sex	Only male mice were used in this study.
Field-collected samples	The study did not involve samples collected from the field.
Ethics oversight	All protocols concerning animal studies were approved by the Institutional Animal Care and Use Committees at Zhejiang University, Boston Children's Hospital and the University of South Florida.

Note that full information on the approval of the study protocol must also be provided in the manuscript.
